# Genotype–phenotype correlation of *BMPR1a* disease causing variants in juvenile polyposis syndrome

**DOI:** 10.1186/s13053-023-00255-3

**Published:** 2023-07-03

**Authors:** M. E. Papadopulos, J. P. Plazzer, F. A. Macrae

**Affiliations:** 1 Department of Medicine, University of Melbourne, The Royal Melbourne Hospital, Melbourne, Australia; 2 Department of Colorectal Medicine and Genetics, The Royal Melbourne Hospital, Melbourne, Australia

**Keywords:** *BMPR1a*, Juvenile Polyposis Syndrome, Juvenile polyps, GI malignancy

## Abstract

**Background:**

Juvenile Polyposis Syndrome (JPS) is an autosomal dominant condition with hamartomatous polyps in the gastrointestinal tract, associated with an increased risk of gastrointestinal malignancy. Disease causing variants (DCVs) in *BMPR1a* or *SMAD4* account for 45–60% of JPS cases, with *BMPR1a* DCVs accounting for 17–38% of JPS cases. Within those with either a *BMPR1a* or *SMAD4* DCV, there is phenotypic variability in location of polyps, risk of malignancy and extra-intestinal manifestations with limited published reports of gene-phenotype association or genotype–phenotype correlation.

We aimed to identify any gene-phenotype association or genotype–phenotype correlation in *BMPR1a* to inform surveillance recommendations, and gene-specific modification to the ACMG classification of pathogenicity of DCVs.

**Methods:**

A literature search was performed through EMBASE, MEDLINE and PubMed. Studies that were included explored *BMPR1a* DCV-related JPS or contiguous deletion of *PTEN* and *BMPR1a*. Data was also drawn from the *BMPR1a* specific databases on LOVD and ClinVar.

**Results:**

There were 211 DCVs in *BMPR1a* identified, 82 from patients with JPS in the literature, and 17 from LOVD and 112 from ClinVar classified as pathogenic or likely pathogenic. These included missense, nonsense and frameshift variants and large deletions, occurring across all functional domains of the gene.

Unlike in *SMAD4* carriers, gastric polyposis and malignancy were not identified in our review in *BMPR1a* carriers, but colonic polyposis and malignancy occurred in carriers of either *BMPR1a* or *SMAD4* DCVs. Those with contiguous deletion of *PTEN* and *BMPR1a* can present with JPS of infancy, with a severe phenotype of GI bleeding, diarrhoea, exudative enteropathy and rectal prolapse.

No specific *BMPR1a* genotype–phenotype correlation could be ascertained including by variant type or functional domain.

**Conclusion:**

Phenotypic characteristics cannot be used to inform variant location in *BMPR1a*. However, the phenotypic characteristics of *BMPR1a* DCV carriers, being almost exclusively related to the colon and rectum, can assist in pathogenicity assessment of *BMPR1a* variants.

Given these findings, we propose that carriers of *BMPR1a* DCVs should only require surveillance for colorectal polyps and malignancy, and that surveillance for gastric polyps and malignancy may be unnecessary. However variant location within *BMPR1a* does not support differential surveillance recommendations.

**Supplementary Information:**

The online version contains supplementary material available at 10.1186/s13053-023-00255-3.

## Background

Juvenile Polyposis Syndrome (JPS) is a rare autosomal dominant condition predisposing to gastrointestinal (GI) hamartomatous polyps. JPS is associated with an increased risk of GI malignancy, with a cumulative lifetime risk of cancer of 38.7% to 86.2% [1, 2].

JPS typically presents with rectal bleeding and anaemia in the second and third decades of life [3, 4], but cases have been reported with presentation during infancy and childhood [5]. Diagnostic criteria for JPS include five or more pathologically defined juvenile polyps in the colon, or at least one pathologically defined juvenile polyp in both the upper and lower GI tract, or any number of juvenile polyps with a family history of JPS [6].

Clinically JPS can be divided into three phenotypic subtypes; Generalised Juvenile Polyposis (GJP) where polyps are seen in the stomach, small intestine, colon and rectum, Juvenile Polyposis Coli (JPC) where polyps are seen only in the colon and rectum, and Juvenile Polyposis of Infancy (JPI) where onset of symptoms occurs at a very young age [7].

The term juvenile relates to the histological type of polyp seen in JPS, rather than the age of onset. Macroscopically, juvenile polyps are lobulated and pedunculated with surface erosion, and microscopically they appear cystic, with dilated glands and inflammatory cells [8].

**Fig. 1:**
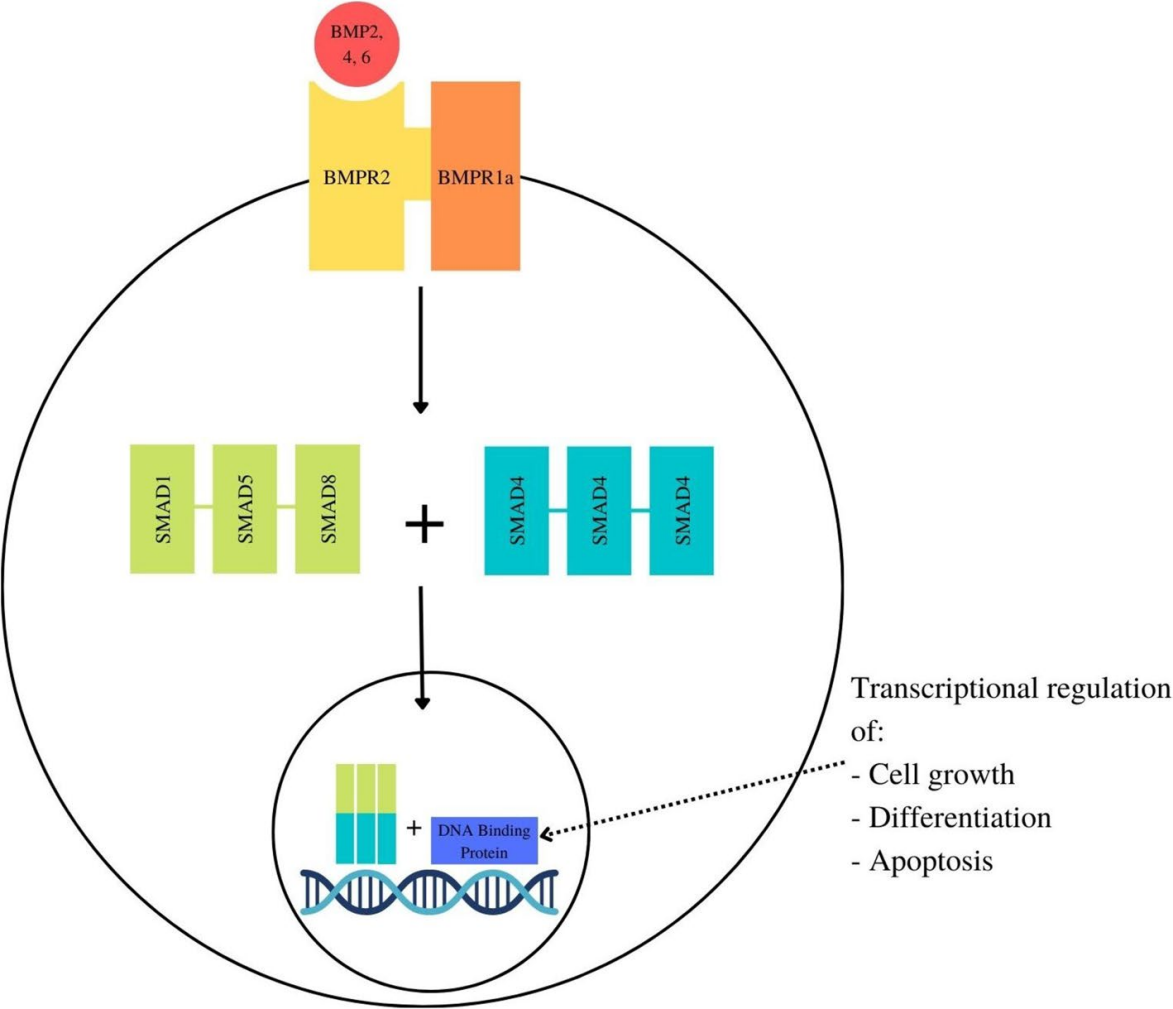
The TGFβ superfamily pathway including intracellular signalling of BMPR1a via SMAD4

Most cases of JPS are caused by disease causing variants (DCVs) of *SMAD4* or *BMPR1a* [9]. Detection of a DCV in *BMPR1a* or *SMAD4* is considered diagnostic of JPS even if the clinical features are inconclusive [10]. The *BMPR1a* gene (10q23.2) comprises 11 coding exons and 1599 nucleotides, which encodes the BMPR1a protein, comprising 533 amino acids, which is a type 1 receptor of the TGFβ superfamily that mediates BMP intracellular signalling through SMAD4 and is involved in colonic epithelial growth [7, 11] (See Fig. 1).

JPS has a highly variable phenotype in terms of location of polyps along the GI tract, number of polyps, age of onset, risk of malignant transformation and extra-intestinal manifestations [12]. The phenotypic variability seen in JPS is only partially explained by possessing either a *BMPR1a* or *SMAD4* DCV [4, 13]. Additionally, there is a group of those with JPS without a known genetic cause who display the JPS phenotype [14].

A gene-phenotype association, which relates the genotypic differences seen in these patients to the phenotype they display, has been defined, as colorectal polyps are seen in those with either a *BMPR1a* or *SMAD4* DCV, but gastric and upper GI polyps are not commonly seen in those with a *BMPR1a* DCV [4, 13, 15]. It is also widely recognised that only those with a *SMAD4* DCV have Hereditary Haemorrhagic Telangiectasia (HHT) [4].

However, an association has not been drawn between genotype and the three phenotypic subtypes of JPS. Further defining a gene-phenotype association in JPS will be of clinical importance as it may inform surveillance guidelines for carriers of *BMPR1a* DCVs.

A genotype–phenotype correlation, relating to the types and sites of DCVs in *BMPR1a*, and the correlated phenotype displayed by those who harbour such DCVs has not been identified for *BMPR1a* either.

Databases for documenting variants of genes include ClinVar, Leiden Open Variation Database (LOVD), Online Mendelian Inheritance in Man and Human Gene Mutation Database. Variants can now be classified as pathogenic, likely pathogenic, uncertain significance (VUS), likely benign or benign according to The American College of Medical Genetics (ACMG) criteria. However, classification and interpretation of the pathogenicity of variants can be discordant between and within databases due to use of alternate classification criteria, incomplete or variable ascertainment of clinical and other information required to reach a classification or misrepresentation of the ACMG criteria. This creates a problem in clinical practice for prognostication and genetic counselling of patients and families with these genetic variants including cascade testing within families. Through better understanding of the relationship between pathogenic variants and phenotype in JPS, it may be possible to reassign some VUSs or discordant interpretations more definitively. It may also inform current efforts to modify the ACMG criteria for pathogenicity of variants in *BMPR1a* by the InSiGHT ClinGen Variant Curation Expert Panel by adopting gene and disease specific features within such modified criteria.

This review will focus on JPS caused by *BMPR1a* DCVs. The aim of this review is to evaluate the proportion of JPS that is accounted for by DCVs in *BMPR1a* and to identify the gene-specific phenotype associations of carriers of *BMPR1a* DCVs and any genotype–phenotype correlations relating to sites and types of DCVs within the gene.

## Methods

To collect studies for this narrative review, a literature search was performed on 26/7/2021 using the search databases MEDLINE, EMBASE and PubMed. The research strategy used included the key words of JPS, juvenile polyps, hamartomatous polyposis syndrome, *BMPR1a* and 10q23del (see Additional File 1). Articles were deemed to be relevant on the basis of inclusion of *BMPR1a* and JPS in the paper. Hence, the Boolean operator of AND was used, in order to refine the search more specifically to the objectives of this review. Studies were limited to English and in humans, but were not restricted by year.

A search of the literature identified a total of 351 studies across 3 databases (MEDLINE = 96, EMBASE = 155, PubMed = 100). After removal of duplicates, there were 165 studies remaining to be screened. Studies were excluded if they only explored *SMAD4* DCV-related JPS, other hamartomatous polyposis syndromes and other GI polyposis syndromes. Studies were included if they explored *BMPR1a* DCV-related JPS or contiguous deletion of *PTEN* and *BMPR1a*. Abstract screening excluded 73 studies, leaving 92 to be full text screened. These studies were evaluated on the basis of author, year, sample size, study design, aims and objectives, results and limitations. Retrospective studies, prospective studies, case reports and systematic reviews were included. As a result, 44 studies were included in the final review (see Additional Files 2 and 3). Data was also drawn from the *BMPR1a* specific databases on LOVD and ClinVar.

## Results

### Types and locations of DCVs

**Fig. 2:**
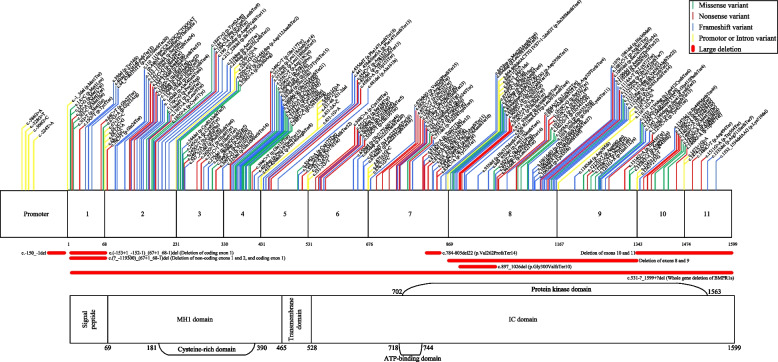
Location and type of DCVs in *BMPR1a* identified in the literature and pathogenic or likely pathogenic variants from LOVD and ClinVar [3, 9, 10, 14, 16–34]

A total of 211 DCVs in *BMPR1a* were identified, 82 from the literature, 17 from LOVD and 112 from ClinVar classified as pathogenic or likely pathogenic (see Additional File 4). 178 of these DCVs occur in coding regions, and 33 occur in non-coding regions of the gene. The DCVs include missense, nonsense and frameshift variants, as well as large deletions (see Fig. 2). Missense variants classified as DCVs were included if they were identified from the literature in patients with JPS, or from LOVD or ClinVar and classified as likely pathogenic or pathogenic by submitters to the respective databases. At this stage, assessment by these submitters has been completed prior to any consideration by the InSiGHT ClinGen Variant Curation Expert Panel based on gene specific modifications of the ACMG criteria.

Of the DCVs occurring in *BMPR1a*, frameshift DCVs predominated (38.86%), followed by nonsense DCVs (24.17%), then missense DCVs (18.01%), with large deletions being the least common (2.80%). DCVs were seen across all functional domains of the gene, with most occurring in the Intracellular domain (46.92%) and the MH1 domain (29.38%). Across all functional domains, frameshift DCVs were the most common (see Table 1). There was no apparent genotype–phenotype correlation.

**Table 1:** Frequency of DCV types across functional domains of *BMPR1a*

	**Missense**	**Nonsense**	**Frameshift**	**Large Deletion**	**Total**	% Total
**Promoter**	**-**	-	-	-	**5**	2.37%
**Intron**	**-**	-	-	-	**28**	13.23%
**Signal Peptide**	**5**	**3**	**3**	**1**	**13**	6.16%
**MH1 Domain**	**15**	**14**	**33**	**0**	**62**	29.38%
Cysteine Rich Domain	14	6	18	0	38	
**Transmembrane Domain**	**1**	**1**	**1**	**0**	**3**	1.42%
**Intracellular Domain**	**17**	**33**	**45**	**4**	**99**	46.92%
Protein Kinase Domain	17	27	38	4	86	
ATP Binding Domain	0	3	2	0	5	
**Whole Gene Deletio**n				**1**	**1**	0.47%
**Total**	**38**	**51**	**82**	**6**	**211**	
% Total	18.01%	24.17%	38.86%	2.80%		

### Proportion of JPS accounted for by BMPR1a DCVs

The proportion of JPS cases accounted for by DCVs in either *BMPR1a* or *SMAD4* is 45% to 60% [16, 35] with *BMPR1a* DCVs accounting for 17% to 38% of cases [3, 10, 16–20].

However, there are still a number of JPS cases with no identifiable DCV, which could be attributed to additional genes which have not yet been identified. MacFarland et al. [14] proposed any additional genes are likely to be low penetrant autosomal dominant or autosomal recessive, due to the lack of family history and younger age at diagnosis seen in the DCV negative group. Alternatively, epigenetic changes may be responsible for driving the phenotype in JPS patients without an identified pathogenic variant [14].

Other reasons could include older studies using more limited gene sequencing techniques which did not detect large deletions. The use of Multiplex Ligation-dependent Probe Amplification (MLPA) has allowed for identification of large deletions in *BMPR1a* causing JPS that were not detected with Sanger sequencing techniques in the past [19, 21]. Contemporarily, Next Generation Sequencing almost always detects both point variants and large deletions. This is demonstrated in a cohort where Sanger sequencing identified 40.9% of JPS cases to have a DCV in either *BMPR1a* or *SMAD4*, while Next Generation Sequencing identified 61% [35].

There may still be cryptic mutations in these genes causing JPS, undetectable to any current gene sequencing technologies.

### Gene-specific phenotype associations

#### Age of diagnosis

DCV negative JPS cases have a significantly lower mean age of diagnosis compared to those carrying either a *BMPR1a* or *SMAD4* DCV (13.1 years vs 21.4 years, *p* = 0.05) [3]. MacFarland et al. found the median age of diagnosis in a selected paediatric population without an identified DCV was 5 years compared to 18 years in those with an identified DCV (*p* < 0.001) [14]. The mean age of diagnosis was similar and not statistically significant for *BMPR1a* compared to *SMAD4* DCV carriers (24.5 years and 28.0 years, respectively) [4, 14, 21].

#### Location of polyps

The location of polyps in *BMPR1a* DCV carriers are predominantly colorectal [15]. Gastric polyps are significantly less common in*BMPR1a* DCV carriers than *SMAD4* DCV carriers (8% vs 73%, *p* < 0.001 [21], and 13% vs 39%, *p* = 0.001 [4]). Similarly, family history of upper GI polyps is significantly lower in *BMPR1a* DCV carriers compared to *SMAD4* DCV carriers (10% vs 85–86%, *p* < 0.01) [3]. In a European retrospective study, polyps in the small intestine were even less common in *BMPR1a* DCV carriers (3.2%) compared to *SMAD4* DCV carriers (15.7%) [4].

#### Number of polyps

Colonic polyps are seen in both carriers of *BMPR1a* and *SMAD4* DCVs [21], however *SMAD4* DCV carriers have higher colorectal polyp numbers than *BMPR1a* DCV carriers [15].

*BMPR1a* DCV carriers do not display massive gastric polyposis (> 100 gastric polyps) [17], with 86% of *BMPR1a* DCV carriers having < 5 gastric polyps, whereas 17% of *SMAD4* DCV carriers had > 100 gastric polyps (*p* = 0.0001) [4].

There was no significant difference in those required to have a colectomy for colorectal polyp control between *BMPR1a* and *SMAD4* DCV carriers. However there was a significant difference in need for colectomy between those with an identified DCV compared to those without an identified DCV (33% vs 3.1%, *p* = 0.03) [14]. There were no *BMPR1a* DCV carriers who required gastrectomy for gastric polyp control or gastric cancer [14].

#### Type of polyps

While juvenile polyps are the most common type of polyp seen in JPS, other polyp types have been reported, including hyperplastic, adenomatous and pseudo-polyps [13, 21–23]. This mixed polyposis is seen in *BMPR1a* DCV carriers but not in *SMAD4* DCV carriers, who typically present with homogenous juvenile polyposis [4].

### Deletion of PTEN/BMPR1a

In addition to large deletions of *BMPR1a* being identified in patients with JPS, cases of JPI have been observed in patients with contiguous deletion of *PTEN* and *BMPR1a*, which often presents in the first two years of life with severe GI bleeding, diarrhoea, exudative enteropathy and rectal prolapse [4, 5, 18, 36–38]. However, there is variability in the phenotype of reported cases, with some presenting later in childhood [39, 40], and some also being associated with dysmorphic features, developmental delay and macrocephaly [5, 41].

Patients with a large deletion of *PTEN* and *BMPR1a* are diagnosed with JPS at a significantly younger age than those who possess a DCV in *BMPR1a* alone (1.5 years vs 23 years, *p* < 0.001) [4].

*PTEN* DCVs cause the PTEN Hamartomatous Tumour Syndromes (PHTS) such as Cowden Syndrome and Bannayan-Riley-Ruvalcaba syndrome, which often present with skin and GI hamartomas, macrocephaly, intellectual disability and developmental delay. The variability in phenotype seen in those with contiguous deletion of both *PTEN* and *BMPR1a* may be explained by heterogeneous and mixed phenotypes of PHTS and JPS.

Additionally, the young age of presentation in contiguous deletion of *PTEN* and *BMPR1a* suggests a synergistic effect of the contiguous gene deletion [5].

### Extra-intestinal manifestations

Extra-intestinal manifestations are not commonly seen in *BMPR1a* DCV carriers. Importantly, those with JPS caused by *BMPR1a* DCVs do not display any features of HHT (epistaxis, telangiectasia and arteriovenous malformations) as seen in *SMAD4* DCV carriers [4].

Congenital heart defects have been reported in *BMPR1a* DCV carriers [4, 10, 42, 43]. In a cohort of patients with JPS, congenital heart defects were observed more in *BMPR1a* than *SMAD4* DCV carriers (9.1% vs 4.2%), however due to limited sample size, this finding was not statistically significant [4].

Other extra-intestinal manifestations seen in *BMPR1a* DCV carriers include facial dysmorphism, macrocephaly, short stature and delayed puberty. Interestingly, these extra-intestinal features are also seen in PHTS, and may have actually been caused by undetected DCVs in *PTEN* rather than *BMPR1a*, due to use of older sequencing techniques.

### Malignancy

JPS is a precancerous condition, with a mean age of diagnosis of 43.9 years and a cumulative lifetime risk at 70 years of age of GI malignancy of 38.7% [1] and for any malignancy of 86.2% [2]. The rates of malignancy reported in cohorts of JPS patients in the literature varies from 11 to 22% [2, 4, 12–15]. Differences in the years of observations between studies accounts for some of the variability in malignancy rates.

In JPS malignant transformation has been thought to occur from permanent mechanical insults, inflammation and repair, following a dysplasia-carcinoma sequence, with cancer arising on a background of generalised mucosal instability, as seen in other hereditary precancerous GI polyposis syndromes such as Familial Adenomatous Polyposis [13, 44].

GI malignancy seen in JPS includes both gastric and colorectal cancers, with colorectal cancer being more common (62% vs 21%) [4] and developing at a younger age than gastric cancer [13]. In a cohort of JPS patients, colorectal cancer was more common than gastric cancer in *BMPR1a* DCV carriers (88% vs 0%) [4]. Indeed, gastric cancer has not been reported in *BMPR1a* DCV carriers [4, 21].

A gene-phenotype association in terms of cancer risk in JPS has been identified, with cancer being less frequently observed in *BMPR1a* DCV carriers than *SMAD4* DCV carriers (8.5% vs 20.5%) [4]. A phenotype associated cancer risk has also been identified, with cumulative risk of malignancy being significantly lower in patients with JPC, than those with GJP (58.7% vs 77.6%, *p* = 0.005) [2].

### Genotype–phenotype correlation

**Fig. 3:**
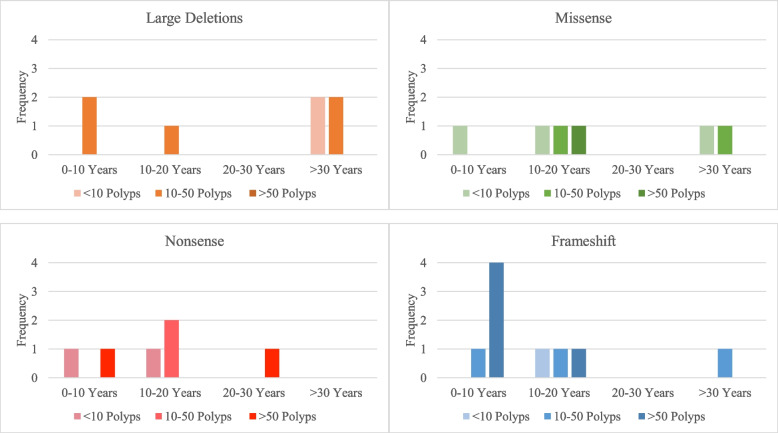
Age of Diagnosis and Number of Colorectal Polyps in patients possessing Large Deletions, Missense, Nonsense or Frameshift DCVs in *BMPR1a*

**Fig. 4:**
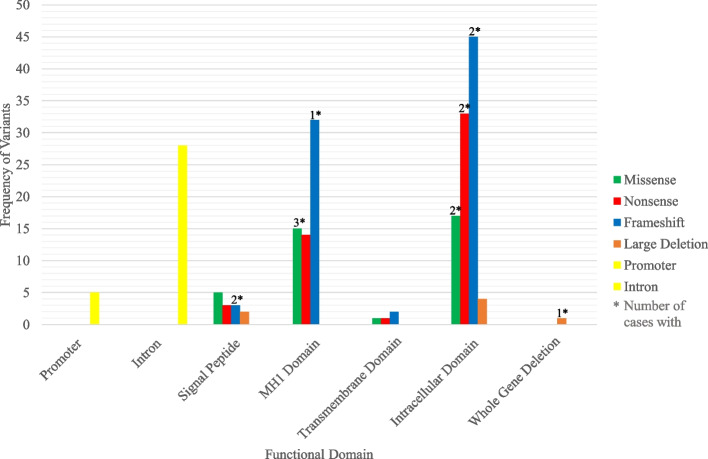
Frequency of DCV type across functional domains of *BMPR1a*, and patients with Colorectal Cancer

Those with DCVs in *BMPR1a* who present at a young age (under 10 years), with high numbers of colorectal polyps (> 50) or who develop colorectal cancer are presumed to carry a DCV which causes a more penetrant phenotype of JPS (see Fig. 3 and 4). Only some DCVs identified from the literature included the accompanying phenotype, compromising the interpretation of a genotype–phenotype correlation from the reported DCVs and penetrance for the phenotype (see Additional File 3).

Amongst patients with large deletions, none were reported to have a high number of colorectal polyps. There were however two patients possessing a large deletion of the entire signal peptide of the *BMPR1a* gene who were diagnosed at a young age (two and eight) with a moderate number of colorectal polyps (10–50) [21].

There were two patients diagnosed at a young age with high numbers of colorectal polyps with nonsense DCVs in the Intracellular Domain; one with the genotype c.1010C > G (p.Ser337Ter), was diagnosed at age six with > 80 colorectal polyps [17, 21], and the other with c.1081C > T (p.Arg361Ter), who was diagnosed with > 150 colorectal polyps, but at a slightly later age of 14 [21].

There were four patients diagnosed at a young age with high numbers of colorectal polyps possessing frameshift DCVs. Two had a duplication in the MH1 Domain: one with a c.351dup (p.Leu118AlafsTer14) was diagnosed at age eight with innumerable colorectal polyps [22], and the other with a c.405dup (p.Pro136ThrfsTer13) who was diagnosed at age seven with 30 colorectal polyps [22]. One had a deletion in the MH1 Domain, c.435delG (p.Phe147LeufsTer18), was diagnosed at age nine with > 50 colorectal polyps [24] and one had a deletion in the Intracellular Domain, c.888delT (p.Gly298ValfsTer10), was diagnosed at age five with > 50 colorectal polyps [17, 21].

There was one patient diagnosed at a young age with a high number of colorectal polyps with a missense DCV in the Intracellular Domain, c.1409 T > C (p.Met770Thr) whose age of diagnosis was not reported, but presented with > 300 colorectal polyps, as well as gastric and small bowel polyps [25].

Young age of diagnosis and high colorectal polyp numbers are not only seen in DCVs in coding regions of *BMPR1a*, as there was one patient with a splice site DCV at intron 4, c.430 + 2 T > C, was diagnosed at age one with > 50 colorectal polyps [21].

Patients with *BMPR1a* DCVs who developed colorectal cancer only possessed DCVs in the coding regions of the gene, including one patient with a whole gene deletion of *BMPR1a*. [20].

Two patients with a DCV in the Signal Peptide Domain at the 5’ end developed colorectal cancer, both with a frameshift DCV, c.44_47delTGTT (p.Leu15SerfsTer20) [20].

In the MH1 Domain, four patients developed colorectal cancer, three with missense DCVs, c.182G > A (p.Cys61Tyr) [14], c.299G > A (p.Cys100Tyr) [23] and c.385 T > A (p.Leu129Ile) [20], and one with a frameshift DCV, c.230 + 452_333 + 41dup (p.Asp112AsnfsTer2) [26].

In the Intracellular Domain, six patients developed colorectal cancer, two with frameshift DCVs, c.665dup (p.Pro223ThrfsTer20) [10] and c.826_827delGA (p.Glu276AsnTer10) [10], two with missense DCVs, c.1127G > A (p.Cys376Tyr) [10] and c.1433G > A (p.Arg478His) [27], and two with nonsense DCVs, c.817C > T (p.Arg273Ter) [10] and c.1081C > T (p.Arg361Ter) [10].

In summary, DCVs causing diagnosis at a young age, high colorectal polyp numbers or colorectal cancer are seen across all functional domains of the gene, and include all DCV types, suggesting a *BMPR1a* genotype–phenotype correlation cannot be identified from the given DCVs in *BMPR1a*.

### Gene-phenotype association

In the literature, a gene-phenotype association has been reported relating to gastric polyposis and gastric cancer being more common in *SMAD4* DCV carriers than *BMPR1a* DCV carriers [4, 13]. Additionally, HHT only occurs in *SMAD4* DCV carriers and is not displayed in *BMPR1a* DCV carriers [4].

While a colonic, age related, or cancer related gene-phenotype association has not yet been reported, there is evidence which should allow for such conclusions to be drawn. The colonic phenotype, JPC, can occur in carriers of either *SMAD4* or *BMPR1a* DCVs, while GJP, affecting the entire gastrointestinal tract appears to only occur in carriers of a *SMAD4* DCV [21]. JPI can occur in carriers of either *BMPR1a* or *SMAD4* DCVs, but will present with a more severe phenotype in those with contiguous deletion of *PTEN* and *BMPR1a* [5]. Colorectal cancer can occur in carriers of either *BMPR1a* or *SMAD4* DCVs but is less common in carriers of *BMPR1a* DCVs than *SMAD4* DCVs, and upper GI malignancy in carriers of *BMPR1a* DCVs does not appear to occur [2, 4, 21].

### Clinical implications of genotype–phenotype correlation

The current surveillance recommendations for JPS state that surveillance for colonic polyps by colonoscopy should commence between ages 12 and 15, or earlier if symptomatic, and be repeated every 1–3 years. Surveillance for gastric polyps by gastroscopy, or small bowel polyps by capsule endoscopy, should commence between ages 12–15, and be repeated every 1–3 years [45]. Once polyps > 10 mm in size are detected, they should be removed [35, 46].

These surveillance recommendations apply to all patients with JPS and do not consider the gene-phenotype association seen in JPS, whereas it has been proposed that surveillance of polyp number, size and malignant transformation based on the phenotypes of GJP or JPC is reasonable [2].

Given that carriers of *BMPR1a* DCVs typically display the JPC phenotype, having low rates of gastric and small intestinal polyps, and that GI malignancy outside the colorectum has not been reported in *BMPR1a* DCV carriers, gastroscopy and capsule endoscopy may not be necessary. These patients may only require monitoring for colorectal polyps and colorectal malignant transformation.

## Conclusion

In the absence of a DCV genotype–phenotype correlation, phenotypic characteristics cannot be used to inform variant location in *BMPR1a*. On current evidence, surveillance of the upper GI tract and small intestine in *BMPR1a* DCV carriers seems unrewarding. Further evaluation of gene-phenotype correlation in JPS is required to confirm this suggestion for clinical surveillance, and evaluate the impact of altered surveillance recommendations.

Reciprocally, the phenotypic characteristics of those with JPS can assist in pathogenicity assessment of DCVs in *BMPR1a*. Accepted *BMPR1a* DCV-positive JPS phenotypes, being largely colonic, that are built on these genotype–phenotype correlations, linked with other specific variants, including VUSs, can then be explored through ancillary studies, such as experimental functional studies, or clinical segregation analyses, offering opportunities for more definitive classification. This may also inform modification of the ACMG criteria for pathogenicity of variants in *BMPR1a*.

There is still a group of JPS patients with no identifiable DCV, which require further genetic analysis to identify additional genes involved, or improved gene sequencing techniques to identify cryptic variants in the current genes identified.

## Supplementary Information

**Additional file 1.** Research strategy and results.

**Additional file 2.** PRISMA diagram.

**Additional file 3.** Table of results.

**Additional file 4.** Table of DCVs and Phenotype identified from literature search and LOVD and ClinVar.

## Data Availability

The datasets generated and/or analysed during the current study are available in the *BMPR1a* Global Variome shared LOVD repository, [https://databases.lovd.nl/shared/variants/BMPR1A] and *BMPR1a* ClinVar repository, [https://www.ncbi.nlm.nih.gov/gene/657].

## Abbreviations

JPS: Juvenile Polyposis Syndrome
GI: Gastrointestinal
GJP: Generalised Juvenile Polyposis
JPC: Juvenile Polyposis Coli
JPI: Juvenile Polyposis of Infancy
DCV: Disease Causing Variant
SMAD4: SMAD Family Member 4
BMPR1a: Bone Morphogenetic Protein Receptor 1a
TGFβ: Transforming Growth Factor β
BMP: Bone Morphogenetic Protein
HHT: Hereditary Haemorrhagic Telangiectasia
LOVD: Leiden Open Variant Database
VUS: Variants of Uncertain Significance
AMCG: American College of Clinical Genetics
InSiGHT: International Society for Gastrointestinal Hereditary Tumours
MLPA: Multiplex Ligation-dependent Probe Amplification
PTEN: Phosphatase and Tensin Homologue
PHTS: PTEN Hamartomatous Tumour Syndromes
