# Supplementary material for: Genotype–phenotype correlation of BMPR1a disease causing variants in juvenile polyposis syndrome
Source: Hered Cancer Clin Pract. 2023 Jul 3;21:12. doi: 10.1186/s13053-023-00255-3 (PMC10316536; doi:10.1186/s13053-023-00255-3)
Supplement: Supplementary file 1 — Additional file 1. Research strategy and results. [file 13053_2023_255_MOESM1_ESM.docx]

**Additional File 1.** Research Strategy and Results

| Search Terms | | Number of Results | | |
| --- | --- | --- | --- | --- |
|  |  | **Pubmed** | **Embase** | **MEDLINE** |
| 1 | Juvenile Polyp* or Juvenile Polyposis Syndrome or JPS or Juvenile Polyposis Coli or Infantile Juvenile Polyposis or Juvenile Polyposis of Infancy or Hamartomatous Polyp* or Hamartomatous Polyposis Syndrome | 1846 | 3043 | 1894 |
| 2 | BMPR1a or Bone Morphogenetic Receptor Type 1A or 10q23del or ACVRLK3 or ALK3 or CD292 or SKR5 | 515 | 1213 | 891 |
| 3 | 1 and 2 | 100 | 175 | 118 |
| 4 | limit 3 to (english language and humans) | 100 | 155 | 96 |
