# Supplementary material for: Genotype–phenotype correlation of BMPR1a disease causing variants in juvenile polyposis syndrome
Source: Hered Cancer Clin Pract. 2023 Jul 3;21:12. doi: 10.1186/s13053-023-00255-3 (PMC10316536; doi:10.1186/s13053-023-00255-3)
Supplement: Supplementary file 2 — Additional file 2. PRISMA diagram. [file 13053_2023_255_MOESM2_ESM.docx]

**Additional File 2.** PRISMA Diagram

Records identified from:

Databases (n = 351)

Registers (n = 0)

Records removed *before screening*:

Duplicate records removed (n = 186)

Records marked as ineligible by automation tools (n = 0)

Records removed for other reasons (n = 0)

Records screened

(n = 165)

Reports excluded:

Other GI Polyposis Syndromes (n = 14)

Other GI Conditions (n = 11)

Only Colorectal Cancer (n = 17)

Only inheritance (n = 3)

Not in humans (n = 9)

In vivo (n = 4)

Not related to JPS (n = 15)

Reports sought for retrieval

(n = 92)

Reports not retrieved

(n = 0)

Reports assessed for eligibility

(n = 92)

Reports excluded:

Only included *SMAD4* related JPS (n = 14)

Cowden Syndrome/PHTS (n = 5)

Hereditary Mixed Polyposis Syndrome (n = 3)

Other GI Polyposis Syndromes (n = 12)

Not evaluating genotype in JPS (n = 14)

Studies included in review

(n = 44)

Reports of included studies

(n = 0)

**Identification of studies via databases and registers**

**Identification**

**Screening**

**Included**
