# Supplementary material for: Genotype–phenotype correlation of BMPR1a disease causing variants in juvenile polyposis syndrome
Source: Hered Cancer Clin Pract. 2023 Jul 3;21:12. doi: 10.1186/s13053-023-00255-3 (PMC10316536; doi:10.1186/s13053-023-00255-3)
Supplement: Supplementary file 3 — Additional file 3. Table of results. [file 13053_2023_255_MOESM3_ESM.docx]

**Additional File 3.** Table of Results

| Author/Year |  | | Method | Results |
| --- | --- | --- | --- | --- |
| Jass et al. (1998) | Title | Juvenile polyposis--a precancerous condition | Clinical and histopathological data from the St. Marks Polyposis Registry was analysed, including age, site and number of juvenile polyps, cancer and type of cancer. | This data allows for a working definition of juvenile polyposis: (1) more than five juvenile polyps of the colorectum; and/or (2) juvenile polyps throughout the gastrointestinal tract; and/or (3) any number of juvenile polyps with a family history of juvenile polyposis. |
|  | Type of Study | Retrospective study |  |  |
|  | Sample Size | 87 patients |  |  |
| Howe et al. (2001) | Title | Germline mutations of the gene encoding bone morphogenetic protein receptor 1A in juvenile polyposis | In the 4 JP kindreds, genotyping was performed using a genome-wide screen. Linkage analysis and mapping was done assuming autosomal dominant inheritance. Simple tandem repeat polymorphisms in BMPR1a were generated and compared to 100 controls. The intron and exon boundaries of BMPR1a were defined using Sequencher, which was used to identify primers. These primers were used in DNA sequencing and mutation analysis in the 4 JP kindreds. Histological samples of polyp tissue from the 4 JP kindreds were microdetected for LOH studies. | In 4 JP kindreds without germline mutations in MADH4 or PTEN, we found germline non-sense mutations in all affected kindred members but not in normal control individuals. These findings indicate involvement of an additional gene in the transforming growth factor-β (TGF-β) superfamily in the genesis of JP, and document an unanticipated function for BMP in colonic epithelial growth control. |
|  | Type of Study | Retrospective study |  |  |
|  | Sample Size | 4 JP Kindreds without MADH4 or PTEN mutation |  |  |
| Zhou et al. (2001) | Title | Germline Mutations in *BMPR1A/ALK3* Cause a Subset of Cases of Juvenile Polyposis Syndrome and of Cowden and Bannayan-Riley-Ruvalcaba Syndromes | Clinical data was analysed, including cancer, cardiac, facial and other extra-intestinal anomalies. Genomic analysis of all 11 coding exons, splice junctions, and flanking in- tronic regions of BMPR1A were examined. LOH analysis was performed. | Overall, 10 (38%) probands were found to have germline BMPR1A mutations, 8 of which resulted in truncated receptors and 2 of which resulted in missense alterations (C124R and C376Y). Almost all available component tumors from mutation-positive cases showed loss of heterozygosity (LOH) in the BMPR1A region, whereas those from mutation-negative cases did not. One proband with CS/CS-like phenotype was also found to have a germline BMPR1A missense mutation (A338D). Thus, germline BMPR1A mutations cause a significant proportion of cases of JPS and might define a small subset of cases of CS/BRRS with specific colonic phenotype. |
|  | Type of Study | Retrospective study |  |  |
|  | Sample Size | 18 unrelated families with JPS and 7 isolated cases of JPS without MADH4 mutation and 21 probands with CS without PTEN mutation |  |  |
| Friedl et al. (2002) | Title | Juvenile polyposis: massive gastric polyposis is more common in MADH4 mutation carriers than in BMPR1A mutation carriers. | Genomic mutation analysis was performed and included all 11 exons and intron boundaries of the MADH4 or BMPR1A genes, respectively, which were then amplified by the polymerase chain reaction (PCR) by using the primers essentially as described by Houlston et al. (1998) and Howe et al. (2001), with small modifications. | MADH4 mutations were identified in seven (24%) and BMPR1A mutations were identified in five patients (17%). A remarkable prevalence of massive gastric polyposis was observed in patients with MADH4 mutations when compared with patients with BMPR1A mutations or without identified mutations. This is the first genotype-phenotype correlation observed in JPS |
|  | Type of Study | Retrospective study |  |  |
|  | Sample Size | 29 unrelated patients with JPS |  |  |
| Sayed et al. (2002) | Title | Germline SMAD4 or BMPR1A mutations and phenotype of juvenile polyposis | Medical data including endoscopic, surgical and pathology reports were reviewed. Patients were asked to complete a JP questionnaire about symptoms, medical history and family history. Histopathological data was reviewed. Genetic analysis was performed using polymerase chain reaction of all exons of SMAD4 and BMPR1A. Products were then sequenced and analysed for mutations | Nine of 54 patients had germline SMAD4 mutations, 13 had BMPR1A mutations, and 32 had neither. There were no significant differences between SMAD4+ and BMPR1A+ cases in terms of clinical factors examined, except for a family history of UGI involvement (P <.01). There was a higher prevalence of familial cases in MUT+ patients (P =.09), >10 lower gastrointestinal polyps (P =.06), and frequency of family history of gastrointestinal cancer compared with MUT- patients (P =.01). Patients with germline SMAD4 or BMPR1A mutations have a more prominent JP phenotype than those without, and SMAD4 mutations predispose to UGI polyposis |
|  | Type of Study | Retrospective study |  |  |
|  | Sample Size | 54 JP probands |  |  |
| Larsen Haidle & Howe (2003, revised 2017) | Title | Juvenile polyposis syndrome | The current literature was reviewed. | Identification of a heterozygous pathogenic variant in SMAD4 or BMPR1A con rms the diagnosis if clinical features are inconclusive. The BMPR1a gene has 11 coding exons and 1599 nucleotides. BMPR1a protein, has 533 amino acids. Type 1 receptor of the TGFb superfamily that mediates BMP intracellular signalling through SMAD4. JPS can be divided into 3 phenotypic subtypes: 1) Generalised Juvenile Polyposis where polyps are seen in the stomach, small intestine, colon and rectum, 2) Juvenile Polyposis Coli where polyps are seen only in the colon and rectum, 3)Juvenile Polyposis of Infancy where onset of symptoms occurs at a very young age.  For individuals following surgical resection: endoscopic evaluation of the rectum and pouch is required. For individuals at risk: monitoring for rectal bleeding and/or anaemia, abdominal pain, constipation, and diarrhea; screening by complete blood count (CBC), colonoscopy, and upper endoscopy starting in the mid- teens (age 15 years) or earlier if symptoms occur. |
|  | Type of Study | Review |  |  |
|  | Sample Size | N/A |  |  |
| Kim et al. (2003) | Title | Identification of a novel BMPR1A germline mutation in a Korean juvenile polyposis patient without SMAD4 mutation | Genetic analysis was performed using PCR and DHPLC, then followed by gene sequencing using bidirectional sequencing. | This patient harboured a novel missense mutation (M470T) in exon 10. In order to rule out the possibility of a polymorphism, 96 unrelated healthy individuals were screened by using direct sequencing, and none was found to show this variation. The patient presented with hematochezia of 1 year's duration and severe anaemia (Hb 7.8 mg/ dl). The patient had approximately 300 polyps throughout the entire gastrointestinal tract, stomach, small bowel, colon and rectum, although the most profuse site was the rectum. A heterotopic pancreas in the proximal jejunum and an accessory spleen in the greater omentum were also found intraoperatively. |
|  | Type of Study | Case report |  |  |
|  | Sample Size | 5 JPS patients without SMAD4 mutation |  |  |
| Howe et al. (2004) | Title | The prevalence of MADH4 and BMPR1A mutations in juvenile polyposis and absence of BMPR2, BMPR1B, and ACVR1 mutations | Genetic analysis was performed using PCR amplification of each exon of five genes (MADH4, BMPR1A, BMPR1B, BMPR2, and/or ACVR1), using primers flanking each intron–exon boundary. Mutations were determined by comparison to wild type sequences using sequence analysis software. | Germline MADH4 mutations were found in 14 cases (18.2%) and BMPR1A mutations in 16 cases (20.8%). No mutations were found in BMPR1B, BMPR2, or ACVR1 in 32 MADH4 and BMPR1A mutation negative cases. This study represents the largest single series of JP patients examined for MADH4 or BMPR1A mutations, and, as such, gives an accurate estimate of the prevalence of these alterations. |
|  | Type of Study | Retrospective study |  |  |
|  | Sample Size | 77 JPS patients |  |  |
| Reichelt et al. (2005) | Title | Juvenile polyposis coli: a facultative precancerosis with some similarities to ulcerative colitis? | Histological specimens were analysed and immunohistochemistry and nuclear staining was performed. | Histological examination showed the typical structure of hamartomatous (juvenile) colorectal polyps consisting of branching glands within an oedematous lamina propria. Focal intraepithelial neoplasia (low-grade) was found in only one of the polyps examined. Cancer in JPS occurs along a dysplasia-carcinoma sequence resulting from permanent mechanical insults, inflammation, and repair rather than from an adenoma-carcinoma sequence as in familial adenomatous polyposis (FAP). |
|  | Type of Study | Case report |  |  |
|  | Sample Size | 1 JPS patient |  |  |
| Delnatte et al. (2006) | Title | Contiguous gene deletion within chromosome arm 10q is associated with juvenile polyposis of infancy, reflecting cooperation between the BMPR1A and PTEN tumour-suppressor genes. | Gene deletion analysis was performed using FISH and PCR. | All patients had germline deletion encompassing two contiguous genes, PTEN and BMPR1A. They all presented in the first months of life with extensive gastrointestinal juvenile hamartomatous polyposis. They all has severe rectal bleeding and macrocephaly. 2 patients presented with facial dysmorphia and 2 patients presented with haemangiomas. |
|  | Type of Study | Case report |  |  |
|  | Sample Size | 4 unrelated JPI patients |  |  |
| Salviati et al. (2006) | Title | Deletion of PTEN and BMPR1A on chromosome 10q23 is not always associated with juvenile polyposis of infancy. | Clinical and histopathological data was retrieved and analysed. A karyotype was performed. FISH was then performed to define the gene deletion. Family members were genotyped. | This patient had a de novo interstitial deletion of chromosome 10q23 that was, however, associated with a significantly milder phenotype. Perinatal and early development were normal. Mild developmental delay was noticed during the 2nd year of life. She had mildly dysmorphic features. Heart sonogram revealed a small atrial septal defect. Our patient had never complained of gastrointestinal symptoms, and she did not have her first episode of mild rectal bleeding until age 5 years. Colonoscopy performed at age 6 years detected multiple (>15) polyps throughout the entire length of the colon. Histology of resected lesions was compatible with juvenile polyps, and the largest polyp also had areas of low-grade focal dysplasia in the head of the lesion. She never experienced major bleeding, iron-deficient anaemia, or diarrhoea. |
|  | Type of Study | Case report |  |  |
|  | Sample Size | 1 patient |  |  |
| Pyatt et al. (2006) | Title | Mutation screening in juvenile polyposis syndrome | Genetic mutations were examined through the sequence analysis of coding regions and exon-intron boundaries in both genes | Germline mutations were identified in 30% of cases, with 11.4% in BMPR1A and 18.6% in MADH4. All mutation-positive individuals were negative for cancer at testing, and a single pulmonary valve stenosis was the only congenital anomaly reported. A majority of mutations identified were novel. |
|  | Type of Study | Retrospective Study |  |  |
|  | Sample Size | 70 JPS patients |  |  |
| Aretz et al. (2007) | Title | High proportion of large genomic deletions and a genotype– phenotype update in 80 unrelated families with juvenile polyposis syndrome | 80 unrelated patients were examined for mutations in the SMAD4, BMPR1A and PTEN genes. 65 had a diagnosis of JPS, 15 had presumed JPS. Gene analysis was performed using direct sequencing of BMPR1a, SMAD4, PTEN and CDH1. Germline mutations were confirmed using PCR. Multiplex Ligation-dependent Probe Amplification (MLPA) was performed to identify large genomic deletions. | In a comprehensive mutation screen of 80 unrelated patients with JPS, we identified point mutations in the SMAD4 and BMPR1A gene in 38% of patients (30/80 families) which is consistent with previous findings,(6,7,20) or in 46% of patients when only the 65 typical cases were considered. By direct sequencing of the two genes, point mutations were identified in 30 patients (46% of typical JPS). Using direct sequencing of individual exons, we identified 17 germline mutations in SMAD4 and 13 mutations in BMPR1A in the 80 patients, resulting in an overall mutation detection rate of 38%, or of 46% when only the unequivocal clinical cases were included. 13 point mutations identified in BMPR1A, five were nonsense, 2 frameshift, 4 missense and 2 splice site mutations. Using MLPA (multiplex ligation-dependent probe amplification assay), large genomic deletions were found in 14% of all patients with typical JPS (six deletions in SMAD4 and three deletions in BMPR1A). Using the recently developed MLPA test kit we identified large SMAD4 and BMPR1A deletions in 26% (9/35) of the remaining mutation-negative patients who fulfilled the clinical diagnostic criteria of JPS.  No probe could be designed for coding exon 5 of the BMPR1A gene because of its high homology to the BMPR1A pseudogene Given the high homology between the BMPR1A gene and a pseudogene, reliability for BMPR1A is not as good as for SMAD4. In particular, wide variability was found with the MLPA probes designed for BMPR1A coding exon 4 and exon 10. |
|  | Type of Study | Retrospective study |  |  |
|  | Sample Size | 80 unrelated JPS patients |  |  |
| Bronsens et al. (2007) | Title | Risk of colorectal cancer in juvenile polyposis | Patient data were collected from The Johns Hopkins Polyposis Registry and clinic. The incidence rates of colorectal cancer in patients with JP were compared with that of the general population through person-year analysis with adjustment for demographics (sex, race and age-specific categories). | The RR (95% CI) for colorectal cancer in patients with JP was 34.0 (14.4 to 65.7). On the basis of an 80-year life span, the absolute risk for colorectal cancer was 38.7 per 100 persons. In this study, the mean age of colorectal cancer diagnosis was 43.9 years with one case being diagnosed at age 30 years. |
|  | Type of Study | Retrospective study |  |  |
|  | Sample Size | 84 JPS patients from 44 families |  |  |
| van Hattem et al. (2008) | Title | Large genomic deletions of SMAD4, BMPR1A and PTEN in juvenile polyposis | Clinical data was collected from The Johns Hopkins Polyposis Registry and two academic hospitals in The Netherlands. Direct sequencing and MLPA analysis were performed to search for germline defects in SMAD4, BMPR1A, PTEN and ENG. | Using direct sequencing and MLPA, a germline defect was detected in 48.1% of JPS patients, including six (22.2%) SMAD4 mutations and three (11.1%) BMPR1A mutations. MLPA identified 14.8% (4/27) of these mutations. |
|  | Type of Study | Retrospective study |  |  |
|  | Sample Size | 29 JPS patients from 27 families |  |  |
| Menko et al. (2008) | Title | Variable phenotypes associated with 10q23 microdeletions involving the PTEN and BMPR1A genes | Chromosome analysis (resolution ~600 bands) and DNA sequence analysis of the PTEN gene were performed. Additionally, fluorescence in situ hybridization (FISH) using different probes for bands 10q23.2 to 10q23.31 and multiplex ligation-dependent probe amplification (MLPA) analysis of the PTEN and BMPR1A genes were performed. The sizes of the deletions were analysed using single nucleotide polymorphism array analysis. | All patients had microdeletions of various sizing, involving both BMPR1a and PTEN. All patients had macrocephaly, dysmorphic features, retardation and congenital abnormalities. One patient developed colorectal cancer. However, only one case had disease onset before 2 years of age and severe symptoms requiring colectomy. No clear correlation was found between ages at onset or severity of gastrointestinal symptoms and the sizes of the deletions. We conclude that patients with 10q23 microdeletions involving the PTEN and BMPR1A genes have variable clinical phenotypes, which cannot be explained merely by the deletion sizes. |
|  | Type of Study | Case report |  |  |
|  | Sample Size | 4 JPI patients |  |  |
| Calva-Cerqueira et al. (2009) | Title | The rate of germline mutations and large deletions of SMAD4 and BMPR1A in juvenile polyposis | DNA was extracted from 102 JPS probands, and each exon and intron–exon boundary of SMAD4 and BMPR1A were sequenced. Coding and non-coding exons of SMAD4 and BMPR1A were screened for deletions with multiplex ligation-dependent probe amplification (MLPA). Probes for three exons of BMPR1A have been shown to be less reliable, however, and there is no probe for exon 7 because it is identical to a pseudogene on chromosome 6. Therefore, some deletions involving BMPR1A could be missed, but this still would not explain the lower rate of deletion found in this study | By sequencing, 20 probands had point mutations of SMAD4 and 22 of BMPR1A. By MLPA, one proband had deletion of most of SMAD4, one of both BMPR1A and PTEN, one of the 5' end of BMPR1A, and another of the 5' end of SMAD4. The overall prevalence of SMAD4 and BMPR1A point mutations and deletions in JPS was 45% in the largest series of patients to date. |
|  | Type of Study | Retrospective study |  |  |
|  | Sample Size | 102 JPS probands |  |  |
| Calva-Cerqueira et al. (2010) | Title | Discovery of the BMPR1A promoter and germline mutations that cause juvenile polyposis | DNA was collected from a large Iowa JP family without germline mutations of BMPR1A or SMAD4 by sequencing. A genome screen was performed using simple tandem repeat poly- morphisms and MLPA analysis. In order to further define the precise deletion, we performed Comparative Genomic Hybridisation (CGH) using DNA from an affected and unaffected member of this family. PCR amplification was performed. 5' RACE studies were performed to define the promoter of BMPR1a. For functional assessment of the identified mutation, site-directed mutagenesis of transcription factor regulatory-binding sites, followed by in Silico analysis was performed. | MLPA analysis revealed a possible deletion in two probes from a NC exon of BMPR1A, and all 10 affected members were found to have this deletion. CGH revealed a heterozygous loss of the 10 probes between 88 515 308 and 88 529 316 on chromosome 10. PCR amplification revealed the location of the mutation was likely the promoter region for BMPR1A.  A total of 6 of 65 JP probands were found to have mutations affecting this promoter. We conclude that we have identified the promoter for BMPR1A, in which mutations may be responsible for as many as 10% of JP cases with unknown mutations. |
|  | Type of Study | Prospective study |  |  |
|  | Sample Size | 10 members of a JPS kindred plus 62 JPS patients |  |  |
| Breckpot et al. (2012) | Title | BMPR1A is a candidate gene for congenital heart defects associated with the recurrent 10q22q23 deletion syndrome | A karyotype was performed, followed by FISH. CGH was performed to identify the location of the deletion. | We report on a de novo intragenic deletion of the BMPR1A gene in a normally developing 17 year old boy with short stature, delayed puberty, facial dysmorphism and an atrioventricular septal defect. |
|  | Type of Study | Case report |  |  |
|  | Sample Size | 1 patient |  |  |
| Latchford et al. (2012) | Title | Juvenile polyposis syndrome: a study of genotype, phenotype, and long-term outcome | Clinical data was reviewed and analysed from The Polyposis Registry, St Mark’s Hospital. Data collected included patient demographics, phenotype, genotype, surveillance performed, and long-term outcomes. Intestinal phenotype was categorized into juvenile polyposis coli, generalized juvenile polyposis, and juvenile polyposis of infancy. All patients were under surveillance, but because this was an historical review covering many years, the surveillance protocol had changed during the study period. | Nineteen patients had SMAD4 mutation and 9 had BMPR1A mutation. Five patients (11%) had valvular heart disease. Six patients (14%) developed cancer; 4 had cancer at the time of diagnosis of juvenile polyposis syndrome, 3 developed cancer while on surveillance. All patients with advanced upper GI disease had SMAD4 mutations. All patients with telangiectasia had SMAD4 mutation. Colonic polyps predominated; 535 of 767 (69.8%) of colonic polyps were right sided. One patient had a solitary significant small-bowel polyp. 65 juvenile polyps contained dysplasia (mild to moderate). Two patients had severe dysplasia or cancer found in carpeting polyps. |
|  | Type of Study | Retrospective study |  |  |
|  | Sample Size | 44 JPS patients from 30 kindreds |  |  |
| Howe et al. (2013) | Title | BMPR1A mutations in juvenile polyposis affect cellular localization | Eight distinct mutations were chosen for analysis. BMPR1A wild-type expression plasmid were tagged with green fluorescent protein on its C-terminus. Site-directed mutagenesis was used to recreate JP patient mutations from the WT-green fluorescent protein BMPR1A plasmid. Direct sequencing confirmed the expression. MPR1A expression vectors were transfected into HEK-293T cells, then confocal microscopy performed to determine cellular localization. | Of the eight selected mutations, one was within the signalling peptide, 4 were within the extracellular domain, and 3 were within the intracellular domain. All 8 mutations had much less membrane and much more intracellular localization. |
|  | Type of Study | Prospective study |  |  |
|  | Sample Size | N/A |  |  |
| Ngeow et al. (2013) | Title | Prevalence of germline PTEN, BMPR1A, SMAD4, STK11, and ENG mutations in patients with moderate-load colorectal polyps | Medical records were requested to document polyp and cancer history. Pedigrees obtained by a genetic counsellor/physician were also reviewed. All subjects had their polyp phenotypes extracted from available records. Histology slides for polyps from each subject were requested and blindly read by our study gastrointestinal pathologists. DNA was analysed for specific mutations and large rearrangements in PTEN, BMPR1A, SMAD4, and STK11, as well as mutations in ENG. | Of 603 patients, 119 (20%) had a personal history of colorectal cancer and most (461; 76%) had fewer than 30 polyps. Seventy-seven patients (13%) were found to have polyposis-associated mutations, comprising 11 in *ENG* (1.8%), 13 in *PTEN* (2.2%), 13 in *STK11* (2.2%), 20 in *BMPR1A* (3.3%), and 21 in *SMAD4* (3.5%). |
|  | Type of Study | Prospective study |  |  |
|  | Sample Size | 603 patients |  |  |
| Oliveria et al. (2013) | Title | Juvenile polyposis of infancy in a child with deletion of BMPR1A and PTEN genes: surgical approach | DNA was collected for chromosome analysis and FISH. Further genetic testing and array-based comparative genomic hybridization was performed. | The patient is a 6 year old male who has a 3.34 Mb long de novo deletion was identified at 10q23.1q23.31, encompassing the PTEN and BMPR1A genes. The disease course was severe with diarrhea, abdominal pain, inanition, refractory anaemia, rectal bleeding, hypoalbuminemia, and exudative enteropathy. Extra-intestinal features including interatrial communication and patent ductus arteriosus, short stature and digital clubbing were noted. A sub-total colectomy, combined with intraoperative endoscopic removal of ileal and rectal stump polyps, was required for palliative disease control. |
|  | Type of Study | Case report |  |  |
|  | Sample Size | 1 JPI patient |  |  |
| Septer et al. (2013) | Title | Aggressive juvenile polyposis in children with chromosome 10q23 deletion | DNA was collected for karyotyping. Microarray comparative genomic hybridization (aCGH) analysis was performed. | The patient is a boy with a 5.75 Mb de novo deletion of chromosome 10q23 and a 1.03 Mb deletion within chromosome band 1p31.3. He displayed aggressive juvenile polyposis and multiple extra-intestinal anomalies including macrocephaly, developmental delay, short stature, hypothyroidism, atrial septal defect, ventricular septal defect and hypospadias. He required colectomy at six years of age, and early colectomy was a common outcome in other children with similar deletions. Due to the aggressive polyposis and reports of dysplasia and even malignancy at a young age, we propose aggressive gastrointestinal surveillance in children with 10q23 microdeletions encompassing the BMPR1A and PTEN genes to include both the upper and lower gastrointestinal tracts. |
|  | Type of Study | Case report |  |  |
|  | Sample Size | 1 JPI patient |  |  |
| Ellery et al. (2014) | Title | Small intestinal polyp development in 10q23 deletion syndrome | Clinical data was reviewed and analysed. | Both patients presented with dysmorphic features, developmental delay, macrocephaly, and recurrent gastric and colon (juvenile & hyperplastic) polyps. At ages 8 and 12 respectively, both developed small intestinal polyps. |
|  | Type of Study | Case report |  |  |
|  | Sample Size | 2 JPS patients |  |  |
| Yamaguchi et al. (2014) | Title | Identification of coding exon 3 duplication in the BMPR1A gene in a patient with juvenile polyposis syndrome. | Clinical data was collected and reviewed. MLPA was used for deletion detection. | We identified a BMPR1A mutation, which involves a duplication of coding exon 3 (c.230+452_333+441dup1995), on multiple ligation dependent probe amplification in a patient with juvenile polyposis syndrome. The mutation causes a frame- shift, producing a truncated protein (p.D112NfsX2). Therefore, the mutation is believed to be pathogenic. |
|  | Type of Study | Case report |  |  |
|  | Sample Size | 1 JPS patient |  |  |
| Aytac et al. (2015) | Title | Genotype-defined cancer risk in juvenile polyposis syndrome | Patients with SMAD4 or BMPR1a mutations were identified from Inherited Colorectal Cancer Registries. Medical records were reviewed and the clinical patterns of disease were analysed. This information included cancer history, patient characteristics, operations, and gastric and colonic polyp counts. The development of cancer and dysplastic gastrointestinal polyps in any part of the body was noted. | Thirty five patients had germline mutations in either BMPR1A (8 patients) or SMAD4 (27). Colonic phenotype was similar between patients with SMAD4 and BMPR1A mutations, whereas SMAD4 mutations were associated with larger polyp numbers (number of patients with 50 or more gastric polyps: 14 versus 0 respectively). The numbers of patients with rectal polyps was comparable between BMPR1A and SMAD4 mutation carriers (5 versus 17). No patient was diagnosed with cancer in the BMPR1A group, whereas four men with a SMAD4 mutation developed gastrointestinal (3) or extraintestinal (1) cancer. The SMAD4 genotype is associated with a more aggressive upper gastrointestinal malignancy risk in juvenile polyposis syndrome |
|  | Type of Study | Retrospective study |  |  |
|  | Sample Size | 35 JPS patients |  |  |
| Alimi et al. (2015) | Title | Overlap of Juvenile polyposis syndrome and Cowden syndrome due to de novo chromosome 10 deletion involving BMPR1A and PTEN: implications for treatment and surveillance | Clinical records were analysed. Gene analysis was performed using the standard Giemsa method at the time of diagnosis. | The patient is a 23 year old male who was diagnosed with JPS at age 4. He has a de novo large interstitial deletion in the long arm of chromosome 10 [46,XY,del(10)(q22.3q24.1)]. In addition to JPS, he had mild cranio-facial abnormalities, mild tricuspid insufficiency, an umbilical hernia, hypoplastic abdominal oblique muscles, short and broad hands and feet, and developmental delay. |
|  | Type of Study | Case report |  |  |
|  | Sample Size | 1 JPS patient |  |  |
| Chubb et al. (2015) | Title | Genetic diagnosis of high-penetrance susceptibility for colorectal cancer (CRC) is achievable for a high proportion of familial CRC by exome sequencing | Gene sequencing of the mismatch repair genes (MMR) APC, MUTYH, and SMAD4/BMPR1A in 626 early-onset familial CRC cases ascertained through a population-based United Kingdom national registry. | A novel missense mutation in BMPR1a (c.1328G > A) was identified. |
|  | Type of Study | Retrospective study |  |  |
|  | Sample Size | 626 patients |  |  |
| Jelsig et al. (2016) | Title | Germline variants in Hamartomatous Polyposis Syndrome-associated genes from patients with one or few hamartomatous polyps | The gene panel including 26 hamartomatous polyposis-associated genes. Gene analysis was performing using targeted Next Generation Sequencing. The detected germline variants were classified into pathogenicity classes. | We detected several germline variants, among them three in ENG, two in BMPR1A, one in PTEN, and one in SMAD4. Although some of the detected variants have been reported previously none could be definitely pathogenic or likely pathogenic. |
|  | Type of Study | Retrospective study |  |  |
|  | Sample Size | 77 patients with hamartomatous polyps |  |  |
| Jelsig (2016) | Title | Hamartomatous polyps - a clinical and molecular genetic study | The current literature was reviewed. | Hamartomatous polyps (HPs) in the gastrointestinal (GI) tract are rare compared to other types of GI polyps, yet they are the most common type of polyp in children. The symptoms are usually rectal bleeding, abdominal pain, obstipation, anaemia, and/or small bowel obstruction. HPs can be classified as juvenile polyps or Peutz-Jeghers polyps based on their histopathological appearance. |
|  | Type of Study | Review |  |  |
|  | Sample Size | N/A |  |  |
| Takeda et al. (2016) | Title | Magnifying chromoendoscopic and endocytoscopic findings of juvenile polyps in the colon and rectum | Endoscopic images were evaluated in terms of gross appearance, color, pit pattern, surface inflammatory changes and vascularity of polyps. Endocytoscopic images were evaluated with regard to the morphology of glandular cavities, nuclei of glandular cells and interstitial features. | Reddish surfaces (98.1%), surface erosion (92.2%), open pits (90.3%) and low pit density (90.3%) were observed in the majority of JPs by chromoendoscopy. |
|  | Type of Study | Retrospective study |  |  |
|  | Sample Size | 154 Juvenile polyps |  |  |
| Achatz et al. (2017) | Title | Cancer Screening Recommendations and Clinical Management of Inherited Gastrointestinal Cancer Syndromes in Childhood | The current literature was reviewed. | Surveillance recommendations:  -colonic polyps = colonoscopy Starting at age 12–15; every year until no polyps are found, then lengthen interval to every 3 years.  -stomach polyps= gastroscopy Starting at age 15; every 1–2 years.  -small bowel polyps= capsule endoscopy Starting at age 15; every 1–2 years |
|  | Type of Study | Review |  |  |
|  | Sample Size | N/A |  |  |
| Pearlmann et al. (2017) | Title | Prevalence and Spectrum of Germline Cancer Susceptibility Gene Mutations Among Patients With Early-Onset Colorectal Cancer | Clinical data was retrieved and analysed. Next generation sequencing identified genetic mutations. | 3 novel missense mutations in BMPR1a were identified in patients with JPS and early onset CRC. (c.955T>C, c.1058A>G, c.1433G>A) |
|  | Type of Study | Retrospective study |  |  |
|  | Sample Size | 450 patients |  |  |
| Ishida et al. (2018) | Title | Malignant tumors associated with juvenile polyposis syndrome in Japan | The clinical data on JPS cases reported in Japan between January, 1971 and March, 2016. | The sites of malignant tumours were the stomach (n = 31), colorectum (n = 29), small intestine (n = 2), breast (n = 1), and thyroid (n = 1). The lifetime risk (at 70 years) of any malignant tumour was 86.2%. The lifetime risk of gastric cancer was 73.0% and that of colorectal cancer was 51.1%. The risk of these cancers developing was dependent on the type of polyp distribution. |
|  | Type of Study | Review |  |  |
|  | Sample Size | 171 JPS cases |  |  |
| Cohen et al. (2019) | Title | Management of juvenile polyposis syndrome in children and adolescents: A position paper from the Espghan polyposis working group | The current literature was reviewed. | Recommendations for surveillance from the ESPHGAN Polyposis Working Group: 2. Colonoscopic surveillance should commence from age 12 to 15 years, or earlier if symptomatic. Once polyps (>10 mm) are detected they should be removed and colonoscopy repeated annually until polyps >10mm have been resected, then repeated every 1 to 5 years  3. Surveillance of the upper GI tract in affected or at-risk JPS patients is not required in childhood or teenage years, unless there is unexplained anaemia or upper GI symptoms.  5. In JPS patients with an isolated BMPR1A gene mutation, there are no additional investigations required beyond the endoscopic procedures described above. Children with BMPR1A mutation and early onset polyposis and/or a severe phenotype and/or extraintestinal manifestations should be evaluated for PTEN mutation. |
|  | Type of Study | Review |  |  |
|  | Sample Size | N/A |  |  |
| Russell et al. (2019) | Title | Homozygous missense variant in BMPR1A resulting in BMPR signalling disruption and syndromic features | DNA was collected for sequencing. | The patient is a 17 month old female with a homozygous missense variant in BMPR1A (10q23.2 c.1217G > T p.Arg406Leu) causing skeletal abnormalities, growth failure a large atrial septal defect, severe sub- glottic stenosis, laryngomalacia, facial dysmorphisms, and developmental delays. The patient did not display symptoms of JPS, but was young at the time of the report. |
|  | Type of Study | Case report |  |  |
|  | Sample Size | 1 patient |  |  |
| Lieberman et al. (2019) | Title | Variable Features of Juvenile Polyposis Syndrome With Gastric Involvement Among Patients With a Large Genomic Deletion of BMPR1A | Gene analysis was performed using whole-genome sequencing, targeted genotyping, and haplotype analysis. This study defined mutation size, mutation prevalence, and tumour pathogenesis. | All families carried a deletion of 429 kb, encompassing the entire BMPR1A coding sequence and 8 downstream genes. Among carriers, JPS was almost fully penetrant, but clinical features varied widely, ranging from mild to very severe, including pan-enteric polyps, gastritis, and colorectal, oesophageal, and testicular cancer, and carriers with phenotypes, which would not have raised suspicion of JPS. The phenotype in this large cohort was extremely variable, although all carriers shared the same variant and the same genetic background. |
|  | Type of Study | Retrospective study |  |  |
|  | Sample Size | 7 JPS kindreds |  |  |
| Harris et al. (2019) | Title | BMPR1A mutation-positive juvenile polyposis syndrome and atrial septal defect: coincidence or association? | Clinical data was review and analysed. | The patient is a 16-year-old male patient with BMPR1A mutation and incidentally detected atrial septal defect (ASD). |
|  | Type of Study | Case report |  |  |
|  | Sample Size | 1 JPS patient |  |  |
| Blatter et al. (2020) | Title | Disease expression in juvenile polyposis syndrome: a retrospective survey on a cohort of 221 European patients and comparison with a literature-derived cohort of 473 SMAD4/BMPR1A pathogenic variant carriers | A retrospective, questionnaire-based European multicentre survey was performed. A review of established cohorts of SMAD4/ BMPR1A pathogenic variant carriers from the medical literature was also performed. Only patients who fulfilled the clinical criteria for JPS and in whom a disease causing, i.e., likely pathogenic (class 4) or pathogenic (class 5) SMAD4 or BMPR1A germline variant could be identified were submitted. | Compared with BMPR1A carriers, SMAD4 carriers displayed anaemia twice as often (58% vs. 26%), and exclusively showed overlap symptoms with haemorrhagic telangiectasia (32%) and an increased prevalence (39% vs. 13%) of gastric juvenile polyps. Cancer reported in 15% of JPS patients (median age 41 years), mainly occurred in the colorectum (overall: 62%, SMAD4: 58%, BMPR1A: 88%) and the stomach (overall: 21%; SMAD4: 27%, BMPR1A: 0%). |
|  | Type of Study | Retrospective study and review |  |  |
|  | Sample Size | 694 JPS patients |  |  |
| Liu et al. (2020) | Title | Familial juvenile polyposis syndrome with a de novo germline missense variant in BMPR1A gene: a case report. | Germline genetic testing via a multigene panel (66 genes), which included genes associated with hereditary tumours such as APC, BMPR1A, BRCA1, BRCA2, MMR genes, MUTYH, PTEN, SMAD4, STK11, POLD1 and POLE, was performed on the proband and his family members. Sanger sequencing was performed to confirm the mutation. | This patient is a 35 year old male with a germline heterozygous missense variant (c.299G > A) in exon 3 BMPR1A gene. The patient presented with rectal bleeding and was found to have dozens of juvenile and adenomatous polyps. |
|  | Type of Study | Case report |  |  |
|  | Sample Size | 1 JPS patient |  |  |
| Lecoquierre et al. (2020) | Title | Patients with 10q22.3q23.1 recurrent deletion syndrome are at risk for juvenile polyposis | DNA was collected and Next Generation Sequencing was performed. | The patient was a young adult harbouring recurrent deletion of BMPR1a, in a context of intellectual disability, ventricular septal defect and severe juvenile polyposis syndrome diagnosed at the age of 25 years, requiring a surgical preventive colectomy. She developed a gastric adenocarcinoma from which she died at the age of 32. This deletion did not involve PTEN. |
|  | Type of Study | Case report |  |  |
|  | Sample Size | 1 JPS patient |  |  |
| Macfarland et al. (2021) | Title | Phenotypic differences in Juvenile Polyposis Syndrome with or without a disease-causing SMAD4/BMPR1A variant. | Clinical data was analysed, included age at diagnosis, family history, cancer history, need for colectomy/ gastrectomy, and polyp number and location. Patients were only included in the final analysis if a P/LP variant was identified in either SMAD4 or BMPR1A, or if there was documented negative genetic testing for both SMAD4 and BMPR1A, with no P/LP or variant of uncertain significance (VUS) identified in either gene. Patients with a VUS in SMAD4 or BMPR1A were excluded from analysis. No patients were known to have a P/LP variant in any other polyposis gene, however, comprehensive polyposis genetic testing outside of SMAD4 and BMPR1A was not required for inclusion | Compared with DCV-positive JPS, DCV-negative JPS was associated with younger age at diagnosis (P < 0.001), lower likelihood of having a family history of JPS (P < 0.001), and a lower risk of colectomy (P 1⁄4 0.032). Subgroup analysis between SMAD4 and BMPR1A carriers showed that SMAD4 carriers were more likely to have a family history of JPS and required gastrectomy. |
|  | Type of Study | Retrospective study |  |  |
|  | Sample Size | 145 JPS patients |  |  |
| Poaty et al. (2021) | Title | BMPR1A and SMAD4 mutations in juvenile polyposis syndrome: clinicopathological and genetic data from two Congolese patients. | Clinical and histological data was collected. DNA was collected and Next Generation Sequencing was performed. | In 2 Congolese males, a novel deletion in BMPR1a was identified (c.435delG). Histological examination revealed typical JPS features. |
|  | Type of Study | Case report |  |  |
|  | Sample Size | 43 patients |  |  |
