# Supplementary material for: Genotype–phenotype correlation of BMPR1a disease causing variants in juvenile polyposis syndrome
Source: Hered Cancer Clin Pract. 2023 Jul 3;21:12. doi: 10.1186/s13053-023-00255-3 (PMC10316536; doi:10.1186/s13053-023-00255-3)
Supplement: Supplementary file 4 — Additional file 4. Table of DCVs and Phenotype identified from literature search and LOVD and ClinVar. [file 13053_2023_255_MOESM4_ESM.docx]

**Additional File 4.** Table of DCVs and Phenotype identified from literature search and LOVD and ClinVar

| Mutation | Consequence | Exon | Functional Domain | Type of Mutation | LOVD Classification | ClinVar Classification | Age of Dx | FHx JPS | Colonic Polyps | No. Colonic Polyps | Gastric Polyps | CRC | Extra-Intestinal Features |
| --- | --- | --- | --- | --- | --- | --- | --- | --- | --- | --- | --- | --- | --- |
| c. -386G>A | Not reported | N/A | Promoter | Promoter | Not reported | Not reported | Not reported | Not reported | Not reported | Not reported | Not reported | Not reported | Not reported |
| c. -328G>T | Not reported | N/A | Promoter | Promoter | Not reported | Not reported | Not reported | Not reported | Not reported | Not reported | Not reported | Not reported | Not reported |
| c. -306 G>C | Not reported | N/A | Promoter | Promoter | Not reported | Not reported | Not reported | Not reported | Not reported | Not reported | Not reported | Not reported | Not reported |
| c.-224T>A | Not reported | N/A | Promoter | Promoter | Not reported | Not reported | Not reported | Not reported | Not reported | Not reported | Not reported | Not reported | Not reported |
| c.-150_-1del | Not reported | N/A | Promoter | Large Deletion | Not reported | Not reported | 31 | Yes | Yes (JP) | >10 | None | Not reported | None |
|  |  |  |  |  |  |  | 50 | Yes | Yes (JP) | <10 | None | Not reported | None |
|  |  |  |  |  |  |  | 39 | Yes | Yes (JP) | <10 | None | Not reported | None |
|  |  |  |  |  |  |  | 23 | Yes | Yes (JP) | >10 | None | Not reported | None |
|  |  |  |  |  |  |  | 19 | Yes | Yes (JP) | >10 | None | Not reported | None |
|  |  |  |  |  |  |  | 24 | Yes | Yes (JP) | >10 | None | Not reported | None |
|  |  |  |  |  |  |  | 24 | Yes | Yes (JP) | >10 | None | Not reported | None |
|  |  |  |  |  |  |  | 34 | Yes | Yes (JP) | >10 | None | Not reported | None |
| c.-1_2del | p.Met1Ter | N/A | Promoter | Deletion | Not reported | Likely Pathogenic | Not reported | Not reported | Not reported | Not reported | Not reported | Not reported | Not reported |
| c.(-153+1_-152-1)_(67+1_68-1)del | Deletion of exon 1 | Exon 1 | Signal peptide | Large deletion | Pathogenic | Not reported | 8 | Yes | Yes (JP, AP, PP) | >30 | None | Not reported | Not reported |
| c.(?_-119300)_(67+1_68-1)del | Deletion of introns 1 and 2 and exon 1 | Exon 1 | Signal peptide | Large deletion | Pathogenic | Not reported | 2 | Yes | Yes (JP) | 11 | None | Not reported | Not reported |
| c.1A>C | p.Met1Leu | Exon 1 | Signal peptide | Missense | VUS | Pathogenic/ Likely Pathogenic | Not reported | Not reported | Not reported | Not reported | Not reported | Not reported | Not reported |
| c.1A>G | p.Met1Val | Exon 1 | Signal peptide | Missense | Not reported | Pathogenic/ Likely Pathogenic | Not reported | Not reported | Not reported | Not reported | Not reported | Not reported | Not reported |
| c.3G>A | p.Met1Ter | Exon 1 | Signal peptide | Nonsense | Likely pathogenic | Pathogenic | Not reported | Not reported | Not reported | Not reported | Not reported | Not reported | Not reported |
| c.3G>C | p.Met1Ile | Exon 1 | Signal peptide | Missense | Not reported | Pathogenic | Not reported | Not reported | Not reported | Not reported | Not reported | Not reported | Not reported |
| c.3G>T | p.Met1Ile | Exon 1 | Signal peptide | Missense | Not reported | Likely Pathogenic | Not reported | Not reported | Not reported | Not reported | Not reported | Not reported | Not reported |
| c.15C>A | p.Tyr5Ter | Exon 1 | Signal peptide | Nonsense | Not reported | Pathogenic | Not reported | Not reported | Not reported | Not reported | Not reported | Not reported | Not reported |
| c.39del | p.Tyr14fs | Exon 1 | Signal peptide | Deletion | Not reported | Likely Pathogenic | Not reported | Not reported | Not reported | Not reported | Not reported | Not reported | Not reported |
| c.40dup | p.Tyr14LeufsTer15 | Exon 1 | Signal peptide | Duplication | Not reported | Likely Pathogenic | Not reported | Not reported | Not reported | Not reported | Not reported | Not reported | Not reported |
| c.44-47delTGTT | p.Leu15SerfsTer20 | Exon 1 | Signal peptide | Deletion | VUS | Pathogenic | Not reported | Not reported | Yes (JP) | Not reported | Not reported | Not reported | Not reported |
|  |  |  |  |  |  |  | Not reported | Yes | Yes (JP) | >10 | None | FHx CRC | Not reported |
|  |  |  |  |  |  |  | Not reported | Not reported | Yes (JP, HP) | Not reported | Not reported | Yes | Not reported |
|  |  |  |  |  |  |  | Not reported | Not reported | Yes (JP, AP, HP) | Not reported | Not reported | Yes | Not reported |
|  |  |  |  |  |  |  | 68 | Not reported | Yes (JP) | Not reported | Not reported | Not reported | Pancreatic Cancer |
| c.64C>T | p.Gln22Ter | Exon 1 | Signal peptide | Nonsense | Not reported | Pathogenic | Not reported | Not reported | Not reported | Not reported | Not reported | Not reported | Not reported |
| c.67G>A | p.Gly23Arg | Exon 1 | Signal peptide | Missense | Not reported | Likely Pathogenic | Not reported | Not reported | Not reported | Not reported | Not reported | Not reported | Not reported |
| c.67+2T>C | Splice site mutation | Intron 1 | N/A | Splice site mutation | Pathogenic/Likely pathogenic | Not reported | Not reported | Not reported | Not reported | Not reported | Not reported | Not reported | Not reported |
| c.68-3C>G | Splice site mutation | Intron 1 | N/A | Splice site mutation | Pathogenic | Not reported | Not reported | Not reported | Yes (JP) | Not reported | Not reported | Not reported | Adrenal Hamartoma Wilm's tumour  Congenital heart defect |
| c.68-1G>A | Splice site mutation | Intron 1 | N/A | Splice site mutation | Not reported | Pathogenic | Not reported | Not reported | Not reported | Not reported | Not reported | Not reported | Not reported |
| c.68-1G>C | Splice site mutation | Intron 1 | N/A | Splice site mutation | Not reported | Pathogenic | Not reported | Not reported | Not reported | Not reported | Not reported | Not reported | Not reported |
| c.70C>T | p.Gln24Ter | Exon 2 | MH1 domain | Nonsense | Pathogenic | Not reported | Not reported | Not reported | Not reported | Not reported | Not reported | Not reported | Not reported |
| c.110_111insCCATGGCACTGGGAT | p.Asp38_Ser39insHisGlyThrGlyIle | Exon 2 | MH1 domain | Insertion | Not reported | Pathogenic | Not reported | Not reported | Not reported | Not reported | Not reported | Not reported | Not reported |
| c.115_116insA | p.Ser39fs | Exon 2 | MH1 domain | Insertion | Not reported | Likely Pathogenic | Not reported | Not reported | Not reported | Not reported | Not reported | Not reported | Not reported |
| c.127_137del | p.Lys43TrpfsTer24 | Exon 2 | MH1 domain | Deletion | Pathogenic | Pathogenic | Not reported | Not reported | Not reported | Not reported | Not reported | Not reported | Not reported |
| c.131C>G | p.Ser44Ter | Exon 2 | MH1 domain | Nonsense | Pathogenic | Not reported | Not reported | Not reported | Not reported | Not reported | Not reported | Not reported | Not reported |
| c.132A>G | p.Ser44Ter | Exon 2 | MH1 domain | Nonsense | Not reported | Likely benign | Not reported | Not reported | Yes (JP) | Not reported | Not reported | Not reported | Oesophageal Carcinoma |
| c.133_134del | p.Glu45LysfsTer25 | Exon 2 | MH1 domain | Deletion | Pathogenic | Pathogenic | Not reported | Not reported | Not reported | Not reported | Not reported | Not reported | Not reported |
| c.139G>T | p.Gly47Ter | Exon 2 | MH1 domain | Nonsense | Not reported | Pathogenic | Not reported | Not reported | Not reported | Not reported | Not reported | Not reported | Not reported |
| c.143dup | p.Thr49AsnfsTer22 | Exon 2 | MH1 domain | Duplication | Not reported | Likely Pathogenic | Not reported | Not reported | Not reported | Not reported | Not reported | Not reported | Not reported |
| c.150del | p.Ala51HisfsTer9 | Exon 2 | MH1 domain | Deletion | Not reported | Pathogenic | Not reported | Not reported | Not reported | Not reported | Not reported | Not reported | Not reported |
| c.156_165del | p.Glu53CysfsTer4 | Exon 2 | MH1 domain | Deletion | Not reported | Pathogenic | Not reported | Not reported | Not reported | Not reported | Not reported | Not reported | Not reported |
| c.160del | p.Ala54IlefsTer6 | Exon 2 | MH1 domain | Deletion | Not reported | Pathogenic | Not reported | Not reported | Not reported | Not reported | Not reported | Not reported | Not reported |
| c.170C>G | p.Pro57Arg | Exon 2 | MH1 domain | Missense | VUS | Pathogenic | Not reported | Not reported | Not reported | Not reported | Not reported | Not reported | Not reported |
| c.176delT | p.Leu59Ter | Exon 2 | MH1 domain | Deletion | VUS | Pathogenic | Not reported | Not reported | Yes (JP, AP) | Not reported | Not reported | Not reported | Not reported |
| c.176T>A | p.Leu59Ter | Exon 2 | MH1 domain | Nonsense | Not reported | Pathogenic/ Likely Pathogenic | Not reported | Not reported | Not reported | Not reported | Not reported | Not reported | Not reported |
| c.182G>A | p.Cys61Tyr | Exon 2 | MH1 domain (CR) | Missense | Not reported | Not reported | 44 | Not reported | Yes (JP) | Not reported | Not reported | Yes | Not reported |
| c.184T>G | p.Tyr62Asp | Exon 2 | MH1 domain (CR) | Missense | VUS | Not reported | Not reported | Yes | Yes (JP) | >10 | None | Not reported | Not reported |
| c.184_187dup | p.Cys63LeufsTer9 | Exon 2 | MH1 domain (CR) | Duplication | Not reported | Pathogenic | Not reported | Not reported | Not reported | Not reported | Not reported | Not reported | Not reported |
| c.189C>A | p.Cys63Ter | Exon 2 | MH1 domain (CR) | Nonsense | Pathogenic | Pathogenic | Not reported | Not reported | Not reported | Not reported | Not reported | Not reported | Not reported |
| c.199_203dup | p.Asp69ValfsTer11 | Exon 2 | MH1 domain (CR) | Deletion | Not reported | Pathogenic | Not reported | Not reported | Not reported | Not reported | Not reported | Not reported | Not reported |
| c.213_228del | p.Ile72Ter | Exon 2 | MH1 domain (CR) | Deletion | Not reported | Pathogenic | Not reported | Not reported | Not reported | Not reported | Not reported | Not reported | Not reported |
| c.216dup | p.Asn73Ter | Exon 2 | MH1 domain (CR) | Duplication | Not reported | Pathogenic | Not reported | Not reported | Not reported | Not reported | Not reported | Not reported | Not reported |
| c.218dup | p.Asn73LysfsTer2 | Exon 2 | MH1 domain (CR) | Duplication | Pathogenic | Not reported | Not reported | Not reported | Not reported | Not reported | Not reported | Not reported | Not reported |
| c.230+1G>A | Splice site mutation | Intron 2 | N/A | Splice site mutation | Not reported | Likely Pathogenic | Not reported | Not reported | Not reported | Not reported | Not reported | Not reported | Not reported |
| c.231-1G>C | Splice site mutation | Intron 2 | N/A | Splice site mutation | Not reported | Likely Pathogenic | Not reported | Not reported | Not reported | Not reported | Not reported | Not reported | Not reported |
| c.230+452_333+441dup | p.Asp112AsnfsTer2 | Exon 3 | MH1 domain (CR) | Duplication | Pathogenic | Not reported | 31 | Not reported | Yes (JP, AP) | Multiple | Not reported | Yes | Not reported |
| c.233C>T | p.Thr78Ile | Exon 3 | MH1 domain (CR) | Missense | VUS | Conflicting interpretations (Likely Pathogenic/ VUS) | Not reported | Not reported | Not reported | Not reported | Not reported | Not reported | Not reported |
| c.238G>A | p.Gly80Arg | Exon 3 | MH1 domain (CR) | Missense | Pathogenic | Not reported | 43 | Not reported | Yes (JP, AP, PP) | Not reported | Yes (Fundal gland cysts) | Not reported | Not reported |
| c.244del | p.Cys82AlafsTer5 | Exon 3 | MH1 domain (CR) | Deletion | Not reported | Pathogenic | Not reported | Not reported | Not reported | Not reported | Not reported | Not reported | Not reported |
| c.245G>A | p.Cys82Tyr | Exon 3 | MH1 domain (CR) | Missense | VUS | Not reported | Not reported | Not reported | Yes (JP) | >10 | None | Not reported | Not reported |
| c.245G>T | p.Cys82Phe | Exon 3 | MH1 domain (CR) | Missense | Not reported | Likely Pathogenic | Not reported | Not reported | Not reported | Not reported | Not reported | Not reported | Not reported |
| c.246C>A | p.Cys82Ter | Exon 3 | MH1 domain (CR) | Nonsense | Not reported | Pathogenic | Not reported | Not reported | Not reported | Not reported | Not reported | Not reported | Not reported |
| c.247_251del | p.Phe83HisfsTer6 | Exon 3 | MH1 domain (CR) | Deletion | Not reported | Pathogenic | Not reported | Not reported | Not reported | Not reported | Not reported | Not reported | Not reported |
| c.262G>T | p.Glu88Ter | Exon 3 | MH1 domain (CR) | Nonsense | VUS | Pathogenic | Not reported | Yes | Not reported | Not reported | None | Not reported | Not reported |
|  |  |  |  |  |  |  | Not reported | Yes | Not reported | Not reported | Not reported | Not reported | Not reported |
| c.271C>T | p.Gln91Ter | Exon 3 | MH1 domain (CR) | Nonsense | Pathogenic | Pathogenic | Not reported | Not reported | Not reported | Not reported | Not reported | Not reported | Not reported |
| c.275del | p.Gly92GlufsTer5 | Exon 3 | MH1 domain (CR) | Deletion | Not reported | Pathogenic | Not reported | Not reported | Not reported | Not reported | Not reported | Not reported | Not reported |
| c.286_289dup | p.Ala97fs | Exon 3 | MH1 domain (CR) | Duplication | Not reported | Pathogenic | Not reported | Not reported | Not reported | Not reported | Not reported | Not reported | Not reported |
| c.299G>A | p.Cys100Tyr | Exon 3 | MH1 domain (CR) | Missense | VUS | Not reported | 35 | Not reported | Yes (JP, AP) | Dozens | Not reported | Yes | Not reported |
| c.309T>G | p.Tyr103Ter | Exon 3 | MH1 domain (CR) | Nonsense | Not reported | Pathogenic | Not reported | Not reported | Not reported | Not reported | Not reported | Not reported | Not reported |
| c.325del | p.Gln109SerfsTer14 | Exon 3 | MH1 domain (CR) | Deletion | Not reported | Pathogenic | Not reported | Not reported | Not reported | Not reported | Not reported | Not reported | Not reported |
| c.349C>T | p.Gln117Ter | Exon 4 | MH1 domain (CR) | Nonsense | Pathogenic | Not reported | Not reported | Yes | Yes (JP) | >10 | None | Not reported | Not reported |
|  |  |  |  |  |  |  | 13 | Yes | Yes (JP, AP) | 30-50 | None | Not reported | Not reported |
| c.351dup | p.Leu118AlafsTer14 | Exon 4 | MH1 domain (CR) | Duplication | VUS | Not reported | 8 | Not reported | Yes (JP) | Innumerable | Not reported | Not reported | Not reported |
| c.353delT | p.Leu118HisfsTer5 | Exon 4 | MH1 domain (CR) | Deletion | VUS | Not reported | Not reported | Yes | Yes (JP) | Not reported | Yes | Not reported | Not reported |
| c.355C>T | p.Arg119Cys | Exon 4 | MH1 domain (CR) | Missense | Pathogenic | Conflicting interpretations (Likely Pathogenic/ VUS) | 17 | Yes | Yes (JP) | >8 | None | Not reported | Not reported |
| c.359G>C | p.Arg120Pro | Exon 4 | MH1 domain (CR) | Missense | VUS | Not reported | Not reported | Not reported | Yes (JP) | Not reported | Not reported | Not reported | Not reported |
| c.360del | p.Thr121fs | Exon 4 | MH1 domain (CR) | Deletion | Not reported | Pathogenic | Not reported | Not reported | Not reported | Not reported | Not reported | Not reported | Not reported |
| c.366_384del | p.Glu123TyrfsTer15 | Exon 4 | MH1 domain (CR) | Deletion | Not reported | Pathogenic | Not reported | Not reported | Not reported | Not reported | Not reported | Not reported | Not reported |
| c.367del | p.Glu123fs | Exon 4 | MH1 domain (CR) | Deletion | Not reported | Pathogenic | Not reported | Not reported | Not reported | Not reported | Not reported | Not reported | Not reported |
| c.369del | p.Glu123AspfsTer21 | Exon 4 | MH1 domain (CR) | Deletion | Not reported | Pathogenic | Not reported | Not reported | Not reported | Not reported | Not reported | Not reported | Not reported |
| c.370T>C | p.Cys124Arg | Exon 4 | MH1 domain (CR) | Missense | Pathogenic | Pathogenic | Not reported | Not reported | Not reported | Not reported | Not reported | Not reported | Not reported |
| c.371G>A | p.Cys124Tyr | Exon 4 | MH1 domain (CR) | Missense | Not reported | Likely Pathogenic | Not reported | Not reported | Not reported | Not reported | Not reported | Not reported | Not reported |
| c.373T>G | p.Cys125Gly | Exon 4 | MH1 domain (CR) | Missense | VUS | Pathogenic | Not reported | Not reported | Yes (JP, AP) | Not reported | Not reported | Not reported | Not reported |
| c.373del | p.Cys125fs | Exon 4 | MH1 domain (CR) | Deletion | Not reported | Pathogenic | Not reported | Not reported | Not reported | Not reported | Not reported | Not reported | Not reported |
| c.385T>A | p.Leu129Ile | Exon 4 | MH1 domain (CR) | Missense | VUS | Not reported | Not reported | Not reported | Yes (JP, AP) | Not reported | Not reported | Yes | Not reported |
| c.388T>C | p.Cys130Arg | Exon 4 | MH1 domain (CR) | Missense | Pathogenic | Pathogenic | 35 | Not reported | Yes (JP) | >9 | None | Not reported | Not reported |
| c.394C>T | p.Gln132Ter | Exon 4 | MH1 domain | Nonsense | Not reported | Pathogenic | Not reported | Not reported | Not reported | Not reported | Not reported | Not reported | Not reported |
| c.405dup | p.Pro136ThrfsTer13 | Exon 4 | MH1 domain | Duplication | VUS | Pathogenic | 7 | Not reported | Yes (JP) | 30 | Yes | Not reported | Not reported |
| c.419_420delCT | p.Pro140ArgfsTer8 | Exon 4 | MH1 domain | Deletion | Pathogenic | Pathogenic | 20 | Not reported | Yes (JP, HP) | Multiple | None | Not reported | Not reported |
| c.430+1G>A | Splice site mutation | Intron 4 | N/A | Splice site mutation | Not reported | Pathogenic | Not reported | Not reported | Not reported | Not reported | Not reported | Not reported | Not reported |
| c.430+2T>C | Splice site mutation | Intron 4 | N/A | Splice site mutation | Pathogenic | Not reported | 1 | Not reported | Yes (JP, PP) | >50 | None | Not reported | Not reported |
| c.431-66_431-2del | Splice site mutation | Intron 4 | N/A | Splice site mutation | Pathogenic | Not reported | 8 | Yes | Yes (JP, HP) | Multiple | Not reported | Not reported | Not reported |
| c.431-2A>C | Splice site mutation | Intron 4 | N/A | Splice site mutation | Pathogenic | Not reported | Not reported | Not reported | Not reported | Not reported | Not reported | Not reported | Not reported |
| c.431-1G>A | Splice site mutation | Intron 4 | N/A | Splice site mutation | VUS | Not reported | 26 | Not reported | Not reported | Not reported | Not reported | Not reported | Not reported |
| c.435delG | p.Phe147LeufsTer18 | Exon 5 | MH1 domain | Deletion | Not reported | Not reported | 9 | Not reported | Yes (JP) | >50 | Not reported | Not reported | Not reported |
|  |  |  |  |  |  |  | 25 | Not reported | Yes (JP) | >100 | Not reported | Not reported | Not reported |
| c.441_458delinsGA | p.Phe147LeufsTer13 | Exon 5 | MH1 domain | Deletion/Insertion | Not reported | Pathogenic | Not reported | Not reported | Not reported | Not reported | Not reported | Not reported | Not reported |
| c.454C>T | p.Arg152Ter | Exon 5 | MH1 domain | Nonsense | Not reported | Pathogenic | Not reported | Not reported | Not reported | Not reported | Not reported | Not reported | Not reported |
| c.455del | p.Arg152HisfsTer13 | Exon 5 | MH1 domain | Deletion | Not reported | Pathogenic | Not reported | Not reported | Not reported | Not reported | Not reported | Not reported | Not reported |
| c.458G>A | p.Trp153Ter | Exon 5 | MH1 domain | Nonsense | Not reported | Pathogenic | Not reported | Not reported | Not reported | Not reported | Not reported | Not reported | Not reported |
| c.481del | p.Ala161fs | Exon 5 | Transmembrane domain | Deletion | Not reported | Pathogenic | Not reported | Not reported | Not reported | Not reported | Not reported | Not reported | Not reported |
| c.524G>A | p.Cys175Tyr | Exon 5 | Transmembrane domain | Missense | Benign | VUS | 14 | Not reported | Yes (JP) | 1 | Not reported | Not reported | Not reported |
| c.524-525dup | p.Tyr176ValfsTer32 | Exon 5 | Transmembrane domain | Duplication | Pathogenic | Not reported | Not reported | Not reported | Not reported | Not reported | Not reported | Not reported | Not reported |
| c.528C>A | p.Tyr176Ter | Exon 5 | IC domain | Nonsense | VUS | Not reported | Not reported | Not reported | Yes (JP, AP) | Not reported | Not reported | Not reported | Not reported |
| c.531-2A>G | Splice site mutation | Intron 5 | N/A | Splice site mutation | VUS | Not reported | Not reported | Not reported | Not reported | Not reported | Not reported | Not reported | Not reported |
| c.531-1G>T | Splice site mutation | Intron 5 | N/A | Splice site mutation | Pathogenic | Likely Pathogenic | Not reported | Not reported | Yes (JP) | Not reported | Not reported | Not reported | Hypertelorism Macrocephaly |
| c.540C>A | p.Cys180Ter | Exon 6 | IC domain | Nonsense | Not reported | Pathogenic | Not reported | Not reported | Not reported | Not reported | Not reported | Not reported | Not reported |
| c.551C>G | p.Ser184Ter | Exon 6 | IC domain | Nonsense | Not reported | Pathogenic | Not reported | Not reported | Not reported | Not reported | Not reported | Not reported | Not reported |
| c.566dup | p.Tyr189Ter | Exon 6 | IC domain | Duplication | Not reported | Pathogenic | Not reported | Not reported | Not reported | Not reported | Not reported | Not reported | Not reported |
| c.567C>A | p.Tyr189Ter | Exon 6 | IC domain | Nonsense | Not reported | Pathogenic | Not reported | Not reported | Not reported | Not reported | Not reported | Not reported | Not reported |
| c.578dup | p.Leu193PhefsTer5 | Exon 6 | IC domain | Duplication | Not reported | Pathogenic | Not reported | Not reported | Not reported | Not reported | Not reported | Not reported | Not reported |
| c.583C>T | p.Gln195Ter | Exon 6 | IC domain | Nonsense | Pathogenic | Pathogenic | 25 | Yes | Yes (JP) | >70 | Yes | Not reported | Not reported |
| c.650del | p.Gly217ValfsTer44 | Exon 6 | IC domain | Deletion | Not reported | Pathogenic | Not reported | Not reported | Not reported | Not reported | Not reported | Not reported | Not reported |
| c.665dup | p.Pro223ThrfsTer20 | Exon 6 | IC domain | Duplication | Pathogenic | Not reported | Not reported | Not reported | Yes (JP) | Not reported | Not reported | Yes | Hypertelorism Macrocephaly |
| c.673delT | p.Leu225TrpfsTer36 | Exon 6 | IC domain | Deletion | VUS | Not reported | Not reported | Yes | Not reported | Not reported | Not reported | Not reported | Not reported |
| c.674delT | p.Leu225TrpfsTer36 | Exon 6 | IC domain | Deletion | VUS | Pathogenic | 23 | Not reported | Yes (JP, AP, HP) | >5 | Not reported | Not reported | Not reported |
| c.675+1G>C | Splice site mutation | Intron 6 | N/A | Splice site mutation | Not reported | Likely Pathogenic | Not reported | Not reported | Not reported | Not reported | Not reported | Not reported | Not reported |
| c.676-1G>C | Splice site mutation | Intron 6 | N/A | Splice site mutation | Not reported | Likely Pathogenic | Not reported | Not reported | Not reported | Not reported | Not reported | Not reported | Not reported |
| c.676-1G>A | Splice site mutation | Intron 6 | N/A | Splice site mutation | Not reported | Likely Pathogenic | Not reported | Not reported | Not reported | Not reported | Not reported | Not reported | Not reported |
| c.676delG | p.Val226PhefsTer35 | Exon 7 | IC domain | Deletion | Not reported | Likely Pathogenic | Not reported | Not reported | Not reported | Not reported | Not reported | Not reported | Not reported |
| c.682C>T | p.Arg228Ter | Exon 7 | IC domain | Nonsense | Pathogenic | Pathogenic | 18 | Yes | Yes (JP) | 11 | Not reported | Not reported | Congenital heart defect |
|  |  |  |  |  |  |  | Not reported | Not reported | Yes (JP, AP) | Not reported | Not reported | Not reported | Not reported |
| c.697C>T | p.Gln233Ter | Exon 7 | IC domain | Nonsense | VUS | Pathogenic/ Likely Pathogenic | Not reported | Not reported | Yes (JP) | Not reported | Not reported | Not reported | Macrocephaly |
| c.715C>T | p.Gln239Ter | Exon 7 | IC domain (PK) | Nonsense | VUS | Pathogenic | Not reported | Not reported | Yes (JP) | Not reported | Not reported | Not reported | Not reported |
|  |  |  |  |  |  |  | Not reported | Yes | Yes (JP) | Not reported | Not reported | Not reported | Not reported |
|  |  |  |  |  |  |  | Not reported | Yes | Yes (JP) | >10 | None | Not reported | Not reported |
| c.730C>T | p.Arg240Ter | Exon 7 | IC domain (ATP) | Nonsense | Pathogenic | Pathogenic | Not reported | Not reported | Not reported | Not reported | Not reported | Not reported | Not reported |
| c.731dup | p.Tyr245IlefsTer23 | Exon 7 | IC domain (ATP) | Duplication | Not reported | Pathogenic | Not reported | Not reported | Not reported | Not reported | Not reported | Not reported | Not reported |
| c.735T>G | p.Tyr245Ter | Exon 7 | IC domain (ATP) | Nonsense | Not reported | Pathogenic | Not reported | Not reported | Not reported | Not reported | Not reported | Not reported | Not reported |
| c.735_736delinsAT | p.Tyr245Ter | Exon 7 | IC domain (ATP) | Deletion/Insertion | VUS | Likely benign | Not reported | Not reported | Not reported | Not reported | Not reported | Not reported | Not reported |
| c.761G>A | p.Arg254His | Exon 7 | IC domain (PK) | Missense | VUS | VUS | Not reported | Not reported | Not reported | Not reported | Not reported | Not reported | Not reported |
| c.769A>T | p.Lys257Ter | Exon 7 | IC domain (PK) | Nonsense | VUS | Not reported | Not reported | Not reported | Yes (JP, AP) | Not reported | Not reported | Not reported | Not reported |
| c.771del | p.Val258TrpfsTer3 | Exon 7 | IC domain (PK) | Deletion | Not reported | Pathogenic | Not reported | Not reported | Not reported | Not reported | Not reported | Not reported | Not reported |
| c.784-805del22 | p.Val262ProfsTer14 | Exon 7 | IC domain (PK) | Large Deletion | VUS | Not reported | Not reported | Not reported | Yes (JP) | Not reported | Not reported | Not reported | Porphyria Malrotation of the GIT |
| c.812G>A | p.Trp271Ter | Exon 7 | IC domain (PK) | Nonsense | VUS | Pathogenic | Not reported | Not reported | Yes (JP) | Not reported | Not reported | Not reported | Not reported |
|  |  |  |  |  |  |  | Not reported | Yes | Yes (JP) | Not reported | Not reported | Not reported | Not reported |
|  |  |  |  |  |  |  | Not reported | Yes | Yes (JP) | >10 | None | Not reported | Not reported |
| c.813del | p.Trp271CysfsTer12 | Exon 7 | IC domain (PK) | Deletion | Not reported | Pathogenic | Not reported | Not reported | Not reported | Not reported | Not reported | Not reported | Not reported |
| c.813G>A | p.Trp271Ter | Exon 7 | IC domain (PK) | Nonsense | Not reported | Pathogenic | Not reported | Not reported | Not reported | Not reported | Not reported | Not reported | Not reported |
| c.817C>T | p.Arg273Ter | Exon 7 | IC domain (PK) | Nonsense | Pathogenic | Pathogenic | Not reported | Not reported | Yes (JP) | Not reported | Not reported | Yes | Not reported |
| c.824_825del | p.Thr275ArgfsTer11 | Exon 7 | IC domain (PK) | Deletion | Not reported | Pathogenic | Not reported | Not reported | Not reported | Not reported | Not reported | Not reported | Not reported |
| c.826_827delGA | p.Glu276AsnfsTer10 | Exon 7 | IC domain (PK) | Deletion | Pathogenic | Pathogenic | Not reported | Not reported | Yes (JP) | Not reported | Not reported | Yes | Not reported |
| c.834C>G | p.Tyr278Ter | Exon 7 | IC domain (PK) | Nonsense | Not reported | Pathogenic | Not reported | Not reported | Not reported | Not reported | Not reported | Not reported | Not reported |
| c.834C>A | p.Tyr278Ter | Exon 7 | IC domain (PK) | Nonsense | Not reported | Pathogenic | Not reported | Not reported | Not reported | Not reported | Not reported | Not reported | Not reported |
| c.847dup | p.Met283AsnfsTer4 | Exon 7 | IC domain (PK) | Duplication | Not reported | Pathogenic | Not reported | Not reported | Not reported | Not reported | Not reported | Not reported | Not reported |
| c.864dup | p.Leu298ThrfsTer9 | Exon 7 | IC domain (PK) | Duplication | Not reported | Pathogenic | Not reported | Not reported | Not reported | Not reported | Not reported | Not reported | Not reported |
| c.864-868delACTTG IVS7 + 1-2delGT | p.Ile288MetfsTer8 | Exon 7 and Intron 7 | IC domain (PK) | Deletion and Splice site mutation | VUS | Not reported | Not reported | Not reported | Not reported | Not reported | None | Not reported | Not reported |
| c.868+2_868+3del | Splice site mutation | Intron 7 | N/A | Splice site mutation | Pathogenic | Not reported | Not reported | Not reported | Not reported | Not reported | Not reported | Not reported | Not reported |
| c.868+1G>A | Splice site mutation | Intron 7 | N/A | Splice site mutation | VUS | Likely Pathogenic | Not reported | Not reported | Not reported | Not reported | Not reported | Not reported | Not reported |
| c.869-2_871del | Splice site mutation | Intron 7 | N/A | Splice site mutation | Not reported | Likely Pathogenic | Not reported | Not reported | Not reported | Not reported | Not reported | Not reported | Not reported |
| Deletion of Exons 8 and 9 | N/A | Exon 8 and 9 | IC domain (PK) | Large deletion | Not reported | Pathogenic | Not reported | Not reported | Not reported | Not reported | Not reported | Not reported | Not reported |
| c.872T>C | p.Phe291Ser | Exon 8 | IC domain (PK) | Missense | VUS | Not reported | Not reported | Not reported | Yes (JP, HP) | Not reported | Not reported | Not reported | Not reported |
| c.874dup | p.Ile292AsnfsTer6 | Exon 8 | IC domain (PK) | Duplication | Not reported | Pathogenic | Not reported | Not reported | Not reported | Not reported | Not reported | Not reported | Not reported |
| c.878C>T | p.Ala293Val | Exon 8 | IC domain (PK) | Missense | Pathogenic | VUS | Not reported | Not reported | Not reported | Not reported | Not reported | Not reported | Not reported |
| c.884delinsGTTCATAGCGG | p.Asp295fsTer3 | Exon 8 | IC domain (PK) | Deletion/Insertion | Not reported | Pathogenic | Not reported | Not reported | Not reported | Not reported | Not reported | Not reported | Not reported |
| c.888delT | p.Gly298ValfsTer10 | Exon 8 | IC domain (PK) | Deletion | Pathogenic | Not reported | 5 | Not reported | Yes (JP) | >50 | Not reported | Not reported | Not reported |
| c.897_1029del | p.Gly300ThrfsTer20 | Exon 8 | IC domain (PK) | Large deletion | Not reported | Pathogenic | Not reported | Not reported | Not reported | Not reported | Not reported | Not reported | Not reported |
| c.905G>A | p.Trp302Ter | Exon 8 | IC domain (PK) | Nonsense | Not reported | Pathogenic | Not reported | Not reported | Not reported | Not reported | Not reported | Not reported | Not reported |
| c.906G>A | p.Trp302Ter | Exon 8 | IC domain (PK) | Nonsense | Pathogenic | Not reported | Not reported | Not reported | Not reported | Not reported | Not reported | Not reported | Not reported |
| c.910C>T | p.Gln304Ter | Exon 8 | IC domain (PK) | Nonsense | Not reported | Pathogenic | Not reported | Not reported | Not reported | Not reported | Not reported | Not reported | Not reported |
| c.917_920dup | p.Ile308PhefsTer4 | Exon 8 | IC domain (PK) | Duplication | Not reported | Pathogenic | Not reported | Not reported | Not reported | Not reported | Not reported | Not reported | Not reported |
| c.920_921insAATT | p.Leu307LeufsTer4 | Exon 8 | IC domain (PK) | Insertion | VUS | Not reported | Not reported | Not reported | Not reported | Not reported | Not reported | Not reported | Not reported |
| c.935del | p.His312MetfsTer10 | Exon 8 | IC domain (PK) | Deletion | Not reported | Pathogenic | Not reported | Not reported | Not reported | Not reported | Not reported | Not reported | Not reported |
| c.949_952del | p.Leu317MetfsTer4 | Exon 8 | IC domain (PK) | Deletion | Not reported | Pathogenic | Not reported | Not reported | Not reported | Not reported | Not reported | Not reported | Not reported |
| c.957dup | p.Phe320LeufsTer14 | Exon 8 | IC domain (PK) | Duplication | Not reported | Pathogenic | Not reported | Not reported | Not reported | Not reported | Not reported | Not reported | Not reported |
| c.955T>C | p.Leu332Pro | Exon 8 | IC domain (PK) | Missense | VUS | Not reported | Not reported | Not reported | Not reported | Not reported | Not reported | Not reported | Not reported |
| c.961delC | p.Leu321Ter | Exon 8 | IC domain (PK) | Deletion | VUS | Pathogenic | Not reported | Not reported | Yes (JP) | Not reported | Not reported | Not reported | Not reported |
|  |  |  |  |  |  |  | Not reported | Yes | Yes (JP) | Not reported | Not reported | Not reported | Not reported |
|  |  |  |  |  |  |  | Not reported | Yes | Yes (JP) | >10 | None | Not reported | Not reported |
|  |  |  |  |  |  |  | Not reported | Yes | Not reported | Not reported | Not reported | Not reported | Not reported |
| c.964A>T | p.Lys322Ter | Exon 8 | IC domain (PK) | Nonsense | Not reported | Pathogenic | Not reported | Not reported | Not reported | Not reported | Not reported | Not reported | Not reported |
| c.987_992delinsTGTA | p.Arg329SerfsTer4 | Exon 8 | IC domain (PK) | Deletion/Insertion | Not reported | Pathogenic | Not reported | Not reported | Not reported | Not reported | Not reported | Not reported | Not reported |
| c.1010C>G | p.Ser337Ter | Exon 8 | IC domain (PK) | Nonsense | Pathogenic | Not reported | 6 | Yes | Yes (JP) | >80 | Not reported | Not reported | None |
| c.1010C>A | p.Ser337Ter | Exon 8 | IC domain (PK) | Nonsense | Not reported | Pathogenic | Not reported | Not reported | Not reported | Not reported | Not reported | Not reported | Not reported |
| c.1013C>A | p.Ala338Asp | Exon 8 | IC domain (PK) | Missense | Pathogenic | Likely Pathogenic | Not reported | Not reported | Not reported | Not reported | None | Not reported | Not reported |
| c.1021G>T | p.Gly341Cys | Exon 8 | IC domain (PK) | Missense | Not reported | Likely Pathogenic | Not reported | Not reported | Not reported | Not reported | Not reported | Not reported | Not reported |
| c.1058A>G | p.Gln353Arg | Exon 8 | IC domain (PK) | Missense | VUS | VUS | Not reported | Not reported | Not reported | Not reported | Not reported | Not reported | Not reported |
| c.1058_1059dup | p.Gly354LysfsTer11 | Exon 8 | IC domain (PK) | Duplication | Not reported | Pathogenic | Not reported | Not reported | Not reported | Not reported | Not reported | Not reported | Not reported |
| c.1061delG | p.Gly354GlufsTer10 | Exon 8 | IC domain (PK) | Deletion | VUS | Pathogenic | Not reported | Yes | Not reported | Not reported | Not reported | Not reported | Not reported |
| c.1061delGinsCA | p.Gly354AlafsTer27 | Exon 8 | IC domain (PK) | Deletion/insertion | VUS | Not reported | Not reported | Not reported | Not reported | Not reported | Not reported | Not reported | Not reported |
| c.1064delA | p.Lys355SerfsTer9 | Exon 8 | IC domain (PK) | Deletion | VUS | Not reported | Not reported | Not reported | Not reported | Not reported | Not reported | Not reported | Not reported |
| c.1065del | p.Lys355fs | Exon 8 | IC domain (PK) | Deletion | Not reported | Pathogenic | Not reported | Not reported | Not reported | Not reported | Not reported | Not reported | Not reported |
| c.1079_1081del | p.His360del | Exon 8 | IC domain (PK) | Deletion | Pathogenic | Not reported | Not reported | Not reported | Not reported | Not reported | Not reported | Not reported | Not reported |
| c.1081C>T | p.Arg361Ter | Exon 8 | IC domain (PK) | Nonsense | Pathogenic | Pathogenic | Not reported | Not reported | Yes (JP) | Not reported | Not reported | Yes | Not reported |
|  |  |  |  |  |  |  | 14 | Yes | Yes (JP, AP) | >150 | None | Not reported | Not reported |
| c.1095del | p.Ser365ArgfsTer34 | Exon 8 | IC domain (PK) | Deletion | Not reported | Pathogenic | Not reported | Not reported | Not reported | Not reported | Not reported | Not reported | Not reported |
| c.1100del | p.Asn367ThrfsTer32 | Exon 8 | IC domain (PK) | Deletion | Not reported | Pathogenic | Not reported | Not reported | Not reported | Not reported | Not reported | Not reported | Not reported |
| c.1101del | p.Asn367fs | Exon 8 | IC domain (PK) | Deletion | Not reported | Pathogenic | Not reported | Not reported | Not reported | Not reported | Not reported | Not reported | Not reported |
| p.1114A>T | p.Lys372Ter | Exon 8 | IC domain (PK) | Nonsense | Not reported | Pathogenic | Not reported | Not reported | Not reported | Not reported | Not reported | Not reported | Not reported |
| p.1119dup | p.Gly374TrpfsTer7 | Exon 8 | IC domain (PK) | Duplication | Not reported | Pathogenic | Not reported | Not reported | Not reported | Not reported | Not reported | Not reported | Not reported |
| c.1127G>A | p.Cys376Tyr | Exon 8 | IC domain (PK) | Missense | Pathogenic | Pathogenic | Not reported | Not reported | Yes (JP) | Not reported | Not reported | Yes | Congenital heart defect |
| c.1131_1153dup | p.Val385AlafsTer22 | Exon 8 | IC domain (PK) | Duplication | Not reported | Pathogenic | Not reported | Not reported | Not reported | Not reported | Not reported | Not reported | Not reported |
| c.1151dup | p.Val385CysfsTer2 | Exon 8 | IC domain (PK) | Duplication | Not reported | Pathogenic | Not reported | Not reported | Not reported | Not reported | Not reported | Not reported | Not reported |
| c.1152del | p.Val385LeufsTer14 | Exon 8 | IC domain (PK) | Deletion | Not reported | Pathogenic | Not reported | Not reported | Not reported | Not reported | Not reported | Not reported | Not reported |
| c.1166+1G>A | Splice site mutation | Intron 8 | N/A | Splice site mutation | Not reported | Likely Pathogenic | Not reported | Not reported | Not reported | Not reported | Not reported | Not reported | Not reported |
| c.1167-2A>G | Splice site mutation | Intron 8 | N/A | Splice site mutation | Pathogenic | Likely Pathogenic | Not reported | Not reported | Not reported | Not reported | Not reported | Not reported | Not reported |
| c.1167-1G>A | Splice site mutation | Intron 8 | N/A | Splice site mutation | Not reported | Likely Pathogenic | Not reported | Not reported | Not reported | Not reported | Not reported | Not reported | Not reported |
| c.1183del | p.Asp395fs | Exon 9 | IC domain (PK) | Deletion | Not reported | Pathogenic | Not reported | Not reported | Not reported | Not reported | Not reported | Not reported | Not reported |
| c.1217G>T | p.Arg406Leu | Exon 9 | IC domain (PK) | Missense | Not reported | VUS | Not reported | Not reported | Not reported | Not reported | Not reported | Not reported | Skeletal abnormalities Congenital heart defect  Severe sub-glottic stenosis  Laryngomalacia Facial dysmorphism Developmental delay |
| c.1221C>G | p.Tyr405Ter | Exon 9 | IC domain (PK) | Nonsense | Not reported | Pathogenic/ Likely Pathogenic | Not reported | Not reported | Not reported | Not reported | Not reported | Not reported | Not reported |
| c.1229C>T | p.Pro410Leu | Exon 9 | IC domain (PK) | Missense | Pathogenic | Not reported | 14 | Not reported | Yes (JP) | Massive | Not reported | Not reported | Not reported |
| c.1231G>A | p.Glu411Lys | Exon 9 | IC domain (PK) | Missense | VUS | VUS | 8 | Not reported | Yes (JP) | Not reported | Not reported | Not reported | Not reported |
| c.1235_1236dup | p.Leu412CysfsTer6 | Exon 9 | IC domain (PK) | Duplication | Pathogenic | Not reported | Not reported | Not reported | Not reported | Not reported | Not reported | Not reported | Not reported |
| c.1243G>A | p.Glu415Lys | Exon 9 | IC domain (PK) | Missense | VUS | Conflicting interpretations (Benign/Likely Benign/VUS) | Not reported | Not reported | Yes (JP, AP) | Not reported | Not reported | Not reported | Not reported |
| c.1243_1244delinsTTTC | p.Glu415PhefsTer4 | Exon 9 | IC domain (PK) | Deletion/Insertion | Not reported | Likely Pathogenic | Not reported | Not reported | Not reported | Not reported | Not reported | Not reported | Not reported |
| c.1245_1246dup | p.Ser416LysfsTer3 | Exon 9 | IC domain (PK) | Duplication | Not reported | Pathogenic | Not reported | Not reported | Not reported | Not reported | Not reported | Not reported | Not reported |
| c.1267del | p.Gln423SerfsTer13 | Exon 9 | IC domain (PK) | Deletion | Not reported | Pathogenic | Not reported | Not reported | Not reported | Not reported | Not reported | Not reported | Not reported |
| c.1275C>A | p.Tyr425Ter | Exon 9 | IC domain (PK) | Nonsense | VUS | Not reported | 25 | Not reported | Yes (JP) | >5 | Not reported | Not reported | Not reported |
| c.1314G>A | p.Trp438Ter | Exon 9 | IC domain (PK) | Nonsense | Not reported | Pathogenic | Not reported | Not reported | Not reported | Not reported | Not reported | Not reported | Not reported |
| c.1327C>T | p.Arg443Cys | Exon 9 | IC domain (PK) | Missense | VUS | Conflicting interpretations (Benign/Likely Benign/VUS) | Not reported | Not reported | Yes (JP) | >10 | Not reported | Not reported | Not reported |
|  |  |  |  |  |  |  | Not reported | Not reported | Yes (JP, AP) | Not reported | Not reported | Not reported | Not reported |
|  |  |  |  |  |  |  | 7 | Not reported | Yes (JP) | 1 | Not reported | Not reported | Not reported |
| c.1328G>A | p.Arg443His | Exon 9 | IC domain (PK) | Missense | Pathogenic | Not reported | Not reported | Not reported | Not reported | Not reported | Not reported | Not reported | Not reported |
| c.1342+1G>T | Splice site mutation | Intron 9 | N/A | Splice site mutation | Likely pathogenic | Not reported | Not reported | Not reported | Not reported | Not reported | Not reported | Not reported | Not reported |
| c.1342+2T>G | Splice site mutation | Intron 9 | N/A | Splice site mutation | Not reported | Likely pathogenic | Not reported | Not reported | Not reported | Not reported | Not reported | Not reported | Not reported |
| Deletion of exons 10 and 11 | N/A | Exon 10 and 11 | IC domain (PK) | Large deletion | Not reported | Not reported | Not reported | Not reported | Not reported | Not reported | Not reported | Not reported | Not reported |
| c.1347del | p.Ile449MetfsTer49 | Exon 10 | IC domain (PK) | Deletion | VUS | Likely Pathogenic | Not reported | Not reported | Not reported | Not reported | Not reported | Not reported | Not reported |
| c.1360C>T | p.Gln454Ter | Exon 10 | IC domain (PK) | Nonsense | Not reported | Pathogenic | Not reported | Not reported | Not reported | Not reported | Not reported | Not reported | Not reported |
| c.1374C>A | p.Tyr458Ter | Exon 10 | IC domain (PK) | Nonsense | Not reported | Pathogenic | Not reported | Not reported | Not reported | Not reported | Not reported | Not reported | Not reported |
| c.1401C>A | p.Tyr467Ter | Exon 10 | IC domain (PK) | Nonsense | Not reported | Pathogenic | Not reported | Not reported | Not reported | Not reported | Not reported | Not reported | Not reported |
| c.1409T>C | p.Met470Thr | Exon 10 | IC domain (PK) | Missense | Pathogenic | VUS | Not reported | Not reported | Yes (JP) | >300 | Yes and small bowel polyps | Not reported | Not reported |
| c.1433G>A | p.Arg478His | Exon 10 | IC domain (PK) | Missense | VUS | Conflicting interpretations (Likely Benign/VUS) | 26 | Not reported | Yes (JP) | Not reported | Not reported | Yes | Not reported |
| c.1438C>T | p.Arg480Trp | Exon 10 | IC domain (PK) | Missense | Likely Pathogenic | Conflicting interpretations (Pathogenic/ Likely Pathogenic/ VUS) | Not reported | Not reported | Not reported | Not reported | Not reported | Not reported | Not reported |
| c.1460G>A | p.Trp487Ter | Exon 10 | IC domain (PK) | Nonsense | Not reported | Pathogenic | Not reported | Not reported | Not reported | Not reported | Not reported | Not reported | Not reported |
| c.1473+1G>A | Splice site mutation | Intron 10 | N/A | Splice site mutation | Not reported | Likely Pathogenic | Not reported | Not reported | Not reported | Not reported | Not reported | Not reported | Not reported |
| c.1474-2A>G | Splice site mutation | Intron 10 | N/A | Splice site mutation | Not reported | Likely Pathogenic | Not reported | Not reported | Not reported | Not reported | Not reported | Not reported | Not reported |
| c.1480C>T | p.Arg494Ter | Exon 11 | IC domain (PK) | Nonsense | VUS | Pathogenic/ Likely Pathogenic | Not reported | Not reported | Yes (JP, HP) | Not reported | Not reported | Not reported | Not reported |
|  |  |  |  |  |  |  | Not reported | Not reported | Yes (JP) | Not reported | Not reported | Not reported | Uterine fibroids |
| c.1511G>A | p.Trp504Ter | Exon 11 | IC domain (PK) | Nonsense | Not reported | Pathogenic | Not reported | Not reported | Not reported | Not reported | Not reported | Not reported | Not reported |
| c.1533del | p.Arg511SerfsTer5 | Exon 11 | IC domain (PK) | Deletion | Not reported | Likely Pathogenic | Not reported | Not reported | Not reported | Not reported | Not reported | Not reported | Not reported |
| c.1552-1554delAAG | p.Lys519del | Exon 11 | IC domain (PK) | Deletion | Not Reported | Not reported | 30 | Not reported | Yes (JP, AP) | 40 | Yes | Not reported | Congenital heart defect |
| c.531-?_1599+?del | Whole gene deletion of BMPR1a | N/A | N/A | Large Deletion | Not reported | Not reported | Not reported | Not reported | Yes (JP) | Not reported | Not reported | Not reported | Not reported |
|  |  |  |  |  |  |  | Not reported | Not reported | Yes (JP, AP) | Not reported | Not reported | Yes | Thyroid cancer Benign breast disease |
|  |  |  |  |  |  |  | Not reported | Not reported | Yes (JP, HP) | Not reported | Not reported | Not reported | Macrocephaly Developmental delay Benign breast disease |
|  |  |  |  |  |  |  | Not reported | Not reported | Yes (JP) | Not reported | Yes | Not reported | Not reported |

VUS = Variant of Uncertain Significances; CR = Cysteine Rich Domain; PK = Protein Kinase Domain; ATP = ATP Binding Domain; JP = Juvenile Polyps; AP = Adenomatous Polyps; HP = Hyperplastic Polyps; PP = Pseudopolyps.
